# Research landscape, thematic evolution, and translational insights of immune checkpoint inhibitor-induced colitis: a bibliometric analysis (2006-2025)

**DOI:** 10.3389/fimmu.2026.1817557

**Published:** 2026-05-18

**Authors:** Huiyong Zheng, Chuang Lai, Fangteng Liu, Hongliang Luo

**Affiliations:** Department of Gastrointestinal Surgery, The Second Affiliated Hospital, Jiangxi Medical College, Nanchang University, Nanchang, Jiangxi, China

**Keywords:** bibliometrics, colitis, immune checkpoint inhibitors, immune-related adverse events, immunotherapy

## Abstract

**Background:**

In recent years, immune checkpoint inhibitors have been widely adopted in cancer therapy. However, their use is frequently associated with the development of colitis. This study employs bibliometric methods to analyze the knowledge structure and current research trends in immune checkpoint inhibitors induced colitis.

**Methods:**

A systematic literature search was conducted within the Web of Science Core Collection database. Data analysis and visualization were performed using CiteSpace, VOSViewer, and the Bibliometrix package in R software.

**Results:**

The present study collated 1,010 papers on ICI-induced colitis from Web of Science Core Collection, encompassing literature from 62 countries/regions, 1,873 institutions, 7,385 authors, and 373 journals. The United States demonstrated leadership in two key metrics: publication volume, with a total of 470 publications, and total citations, with a total of 41,125 citations. The University of Texas MD Anderson Cancer Center produced the highest number of publications (n=83). Wang Yinghong (n=48) emerged as the most prolific author. The Journal for Immunotherapy of Cancer was the most widely disseminated publication in this field (n=60). An analysis of keywords identified research trends beyond ICI, colitis, and irAE, including Ipilimumab, immunotherapy, Nivolumab, melanoma, cancer, and Pembrolizumab.

**Conclusion:**

This study performed a visual analysis of the fundamental knowledge structure underlying immune checkpoint inhibitors mediated colitis. The results indicate that future research should prioritize the exploration of combination therapies, clinical case management strategies, underlying pathogenic mechanisms, fecal microbiota transplantation, and the identification of predictive and diagnostic biomarkers for adverse events.

## Introduction

Immune checkpoint inhibitors (ICIs) have emerged as a prominent therapeutic modality for various cancers, attributable to their effectiveness in addressing a range of tumor types (1). By targeting cytotoxic T-lymphocyte-associated protein 4 (CTLA-4) or programmed cell death ligand 1 (PD-1/PD-L1) on T cells, ICIs have been shown to enhance antitumor T-cell activity, whether as monotherapy or in combination settings (2, 3). This has led to the observation of durable antitumor responses across a range of tumor types (4–6). However, ICI therapy has been observed to not only amplify immune responses against cancer cells but also to overstimulate the immune system, consequently resulting in immune-related adverse events (irAEs) (7). The incidence of irAEs is influenced by numerous factors, including the type of ICI utilized, the treatment regimen, the cancer type, and patient characteristics (8). These adverse reactions manifest in diverse forms, ranging from mild to severe, and affect multiple organ systems, including colitis, hepatitis, skin toxicity, and endocrine toxicity (9). Among these irAEs, colitis represents one of the most prevalent clinical manifestations. Untreated colitis has the potential to result in severe, life-threatening complications (10). Moreover, effective management strategies for ICI-induced colitis remain inadequate (11). Therefore, it is imperative to develop a comprehensive understanding of the current research landscape, emerging trends, and future directions.

Bibliometrics is a tool that employs various scientometrics analysis software to qualitatively and quantitatively analyze journal development trends, publications within specific fields, and their metadata (12). In contradistinction to conventional research or review methodologies, bibliometrics facilitates systematic and quantitative analysis of publications within discrete domains through the visualization of countries, institutions, authors, keywords, references, papers, journals, and other pertinent elements (13).

To date, a dedicated scientometric analysis focused specifically on ICI-mediated colitis remains lacking. More importantly, ICI-colitis occupies a distinct translational niche within the broader irAE literature because it lies at the intersection of mucosal immunology, steroid-refractory inflammatory toxicity, microbiota-directed intervention, and emerging colitis-specific biomarkers. The objective of this study is to utilize bibliometric analysis to delineate academic contributions, identify the most influential institutions, authors, and journals within this field, and delineate the most significant current research themes and trends. This contributes to the development of safer immunotherapies, the identification of biomarkers, and the refinement of management strategies.

## Methods

### Data retrieval and collection

This study retrieved relevant literature on ICI-induced colitis over the past two decades from the Web of Science Core Collection (WoSCC) on August 7, 2025, and analyzed it using bibliometric methods. The search formula was: TS=((“immune checkpoint inhibitor*” OR “immune checkpoint block*” OR “immune checkpoint therap*” OR “immunological checkpoint inhibitor*” OR “immuno-checkpoint inhibitor*” OR “immune checkpoint blocker*” OR “Anti-CTLA-4” OR “Anti-PD-1” OR “Anti-PD-L1” OR ipilimumab OR tremelimumab OR pembrolizumab OR nivolumab OR avelumab OR atezolizumab OR durvalumab OR cemiplimab OR relatimab OR lambrolizumab OR ticilimumab OR utomilumab OR PD-1 OR PD-L1 OR CTLA-4 OR Immunotherapy OR Immunotherapies OR immunotherapeutic OR yervoy OR Keytruda OR opdivo) AND (colitis OR ulcerative colitis OR “immune-related colitis” OR “checkpoint inhibitor-induced colitis” OR “immunotherapy-associated colitis”)). A total of 2,280 papers were retrieved from the WoSCC database. After further screening for article type and language used in this analysis, and excluding irrelevant literature, we ultimately obtained 1,010 papers for bibliometric analysis. Language was restricted to “English” and article types were limited to ‘article’ and “review”. Publications in plain text format, including full record and cited references were collected and extracted. This scientometric article includes no data or information regarding a patient or trial participant.

### Bibliometric analysis

**Figure 1:**
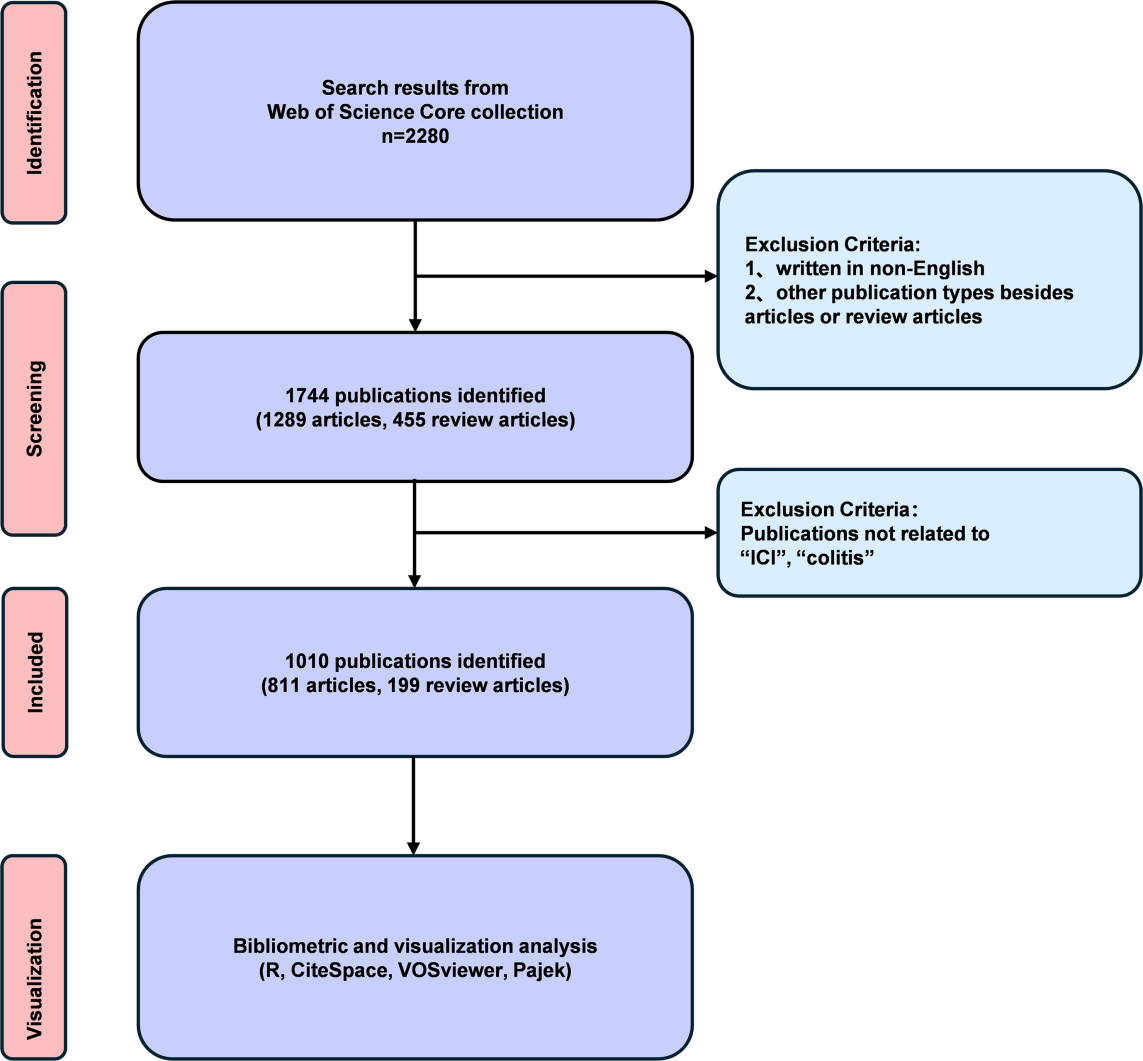
Flowchart of literature data retrieval and processing analysis.

The data processing and visualization were executed using the R programming language (version 4.5.0), the R package bibliometrix (version 5.0.1), the CiteSpace software (version 6.4.R1 Advanced), the VOSviewer (version 1.6.18), and Pajek (version 6.01). The ggplot2 software was employed to analyze article types, maps of corresponding authors’ countries, journal publication volumes, and annual keyword heatmaps. Bibliometrix (14) depicted annual publication volumes by country and institution, three-field maps, thematic evolution maps, topic trend maps, and dendrograms. The CiteSpace 6.4.R1 Advanced (15) software program was utilized to generate a variety of analytical outputs, including co-cited journal maps, journal dual-map overlays, reference cluster maps, reference timeline maps, reference citation burst maps, keyword cluster maps, keyword timeline maps, and keyword citation burst maps. Furthermore, VOSviewer (16) and Pajek were utilized to generate collaboration maps between countries and institutions, author and co-cited author networks, journal density maps, and keyword cluster maps. A detailed study flow chart is provided in **Figure 1**.

### Validation of data source

To mitigate potential single-database bias, a complementary retrieval was conducted in PubMed using identical time span, language, and document-type restrictions. Rather than serving as a direct count-based validation of WoSCC completeness, PubMed was employed as a cross-database sensitivity analysis to assess the reproducibility of major thematic structures across databases.The PubMed search yielded 1,071 records, slightly exceeding the 1,010 records identified in WoSCC, suggesting minor differences in database coverage. Given that WoSCC provides more standardized citation metadata suitable for bibliometric mapping, it was retained as the primary data source for subsequent analyses. Meanwhile, PubMed may include additional clinically oriented or translational studies that are not fully indexed in WoSCC. A comparative analysis of major journals, authors, and high-frequency keywords between the two databases is presented in **Supplementary Material 1**, supporting the overall robustness of the identified research trends.

## Results

### Study characteristics

**Figure 2:**
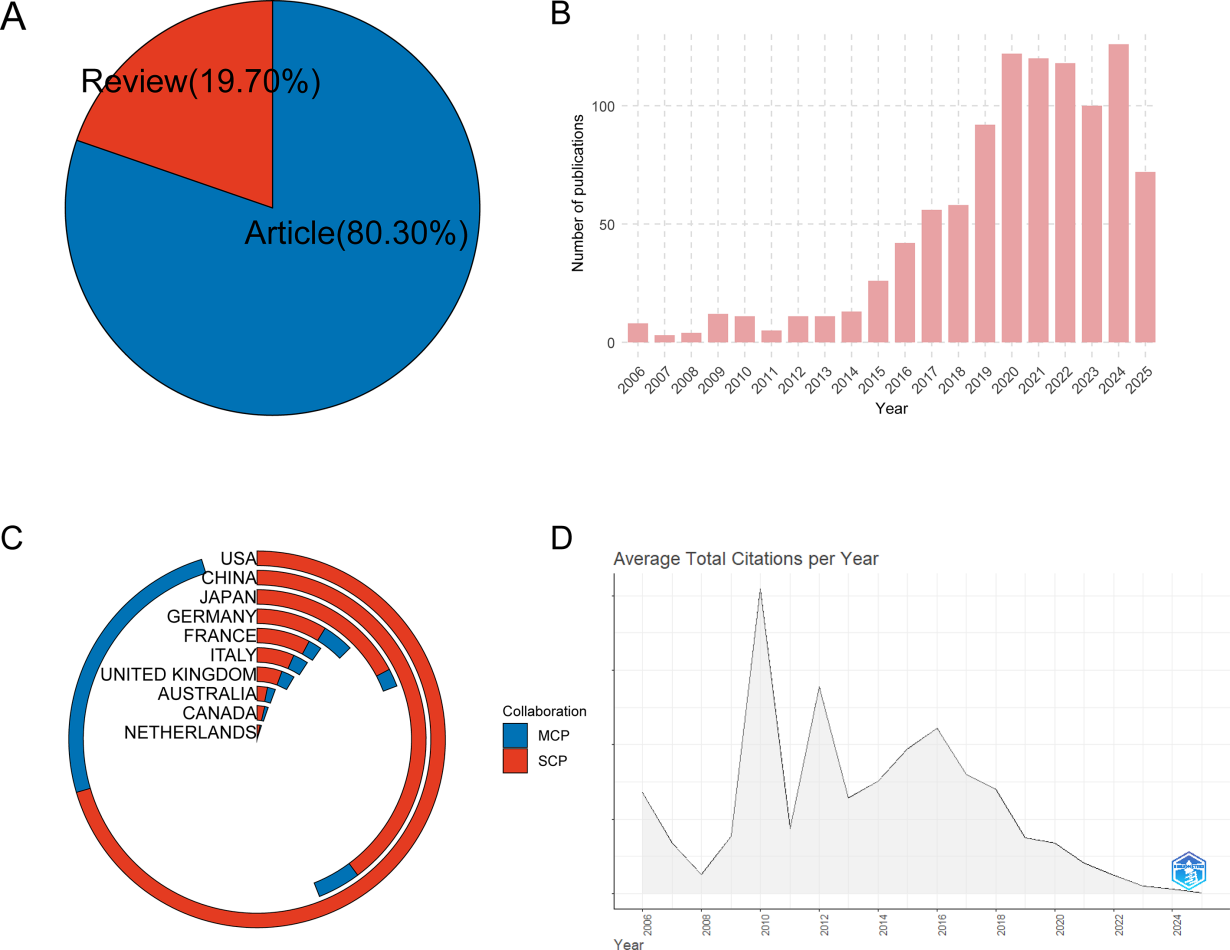
Study characteristics of ICI induced colitis between 2006 and 2025. **(A)** Article type. **(B)** Trend chart of annual publications. **(C)** Corresponding authors’ countries categorized by multi-country publications (MCP) and single-country publications (SCP). **(D)** Average annual citations.

The results demonstrated that between 2006 and August 2025, 1,010 articles pertaining to ICI-induced colitis were retrieved from WoSCC, including 811 articles (80.30%) and 199 reviews (19.70%) (**Figure 2A**). From 2006 to 2025, the annual number of publications exhibited a marked increase (**Figure 2B**). Prior to 2015, the annual publication rate was less than 20, suggesting that the field had received minimal attention and remained largely unexplored. Subsequent to 2015, the number of related publications began to increase rapidly on an annual basis. However, since 2020, the number of publications has exhibited slight fluctuations on an annual basis. An analysis of the countries/regions where the corresponding authors are located (**Figure 2C**) shows that the countries/institutions with the largest number of publications are the United States, China, and Japan. The United States has a high number of publications and a high proportion of MCPs. As illustrated in **Figure 2D**, the average annual citations are presented.

### Country analysis

**Figure 3:**
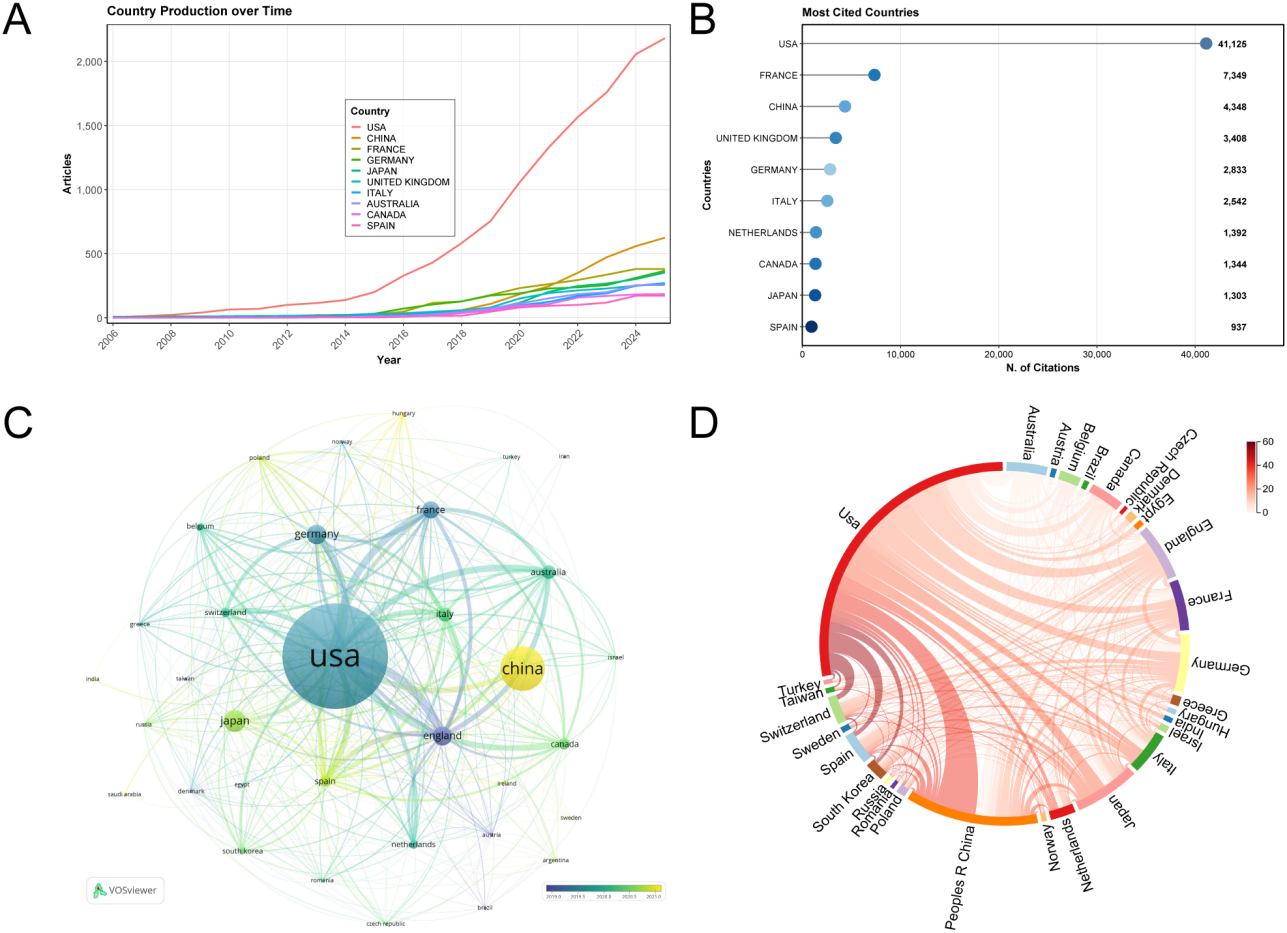
Analysis of countries publishing publications on ICI induced colitis. **(A)** Over time, the top 10 countries with the most published papers. **(B)** Top 10 countries with the most cited times. **(C)** Map of country/region cooperation. **(D)** The chord diagram illustrates the cooperative relationships between countries in the field of colitis research.

A total of 1,010 papers have been published in 62 different countries and regions. As illustrated in **Table 1**, the 20 countries/regions with the highest number of publications on ICI-induced colitis are as follows: The United States leads in terms of the number of publications, with a total of 470 publications, accounting for 46.53% of the total. It is followed by China, with 198 publications, constituting 19.60% of the total, and Japan, with 98 publications, making up 9.70% of the total. The collective number of publications from these three countries accounts for over 75% of the total volume published. The United States has a significantly higher number of articles than other countries, and it has the highest citations and connection strength. It is evident that the United States has published a greater number of articles than other countries, and the growth rate of publication volume is more pronounced (**Figure 3A**). Since 2015, the growth rate has been particularly prominent. As illustrated in **Figure 3B**, the three most frequently cited countries are the United States (n = 41,125), France (n = 7,349), and China (n = 4,348). It is noteworthy that France has a higher average citation, while the average citation of China and Japan is significantly lower than that of other countries, despite both having higher publication volumes. It is possible that the delayed publication of scholarly papers in these two countries has hindered the development of their influence.

**Table 1:** Top 20 most productive countries/regions.

Rank	Country	Documents	Citations	Total link strength
1	Usa	470	51138	476
2	China	198	6728	85
3	Japan	98	5052	104
4	Germany	89	12022	197
5	England	85	14488	285
6	France	77	18447	244
7	Italy	64	9220	221
8	Australia	61	13088	221
9	Canada	51	9729	152
10	Spain	48	10105	247
11	Switzerland	43	5701	144
12	Netherlands	38	7821	120
13	Belgium	32	5140	110
14	South Korea	29	4667	93
15	Greece	15	995	66
16	Poland	14	2593	89
17	Denmark	13	3515	47
18	Israel	11	4380	47
19	Egypt	9	1050	9
20	Sweden	9	647	6

Furthermore, a cooperative network was formulated based on the number of publications and partnerships in each country/region, with 34 countries having a minimum of five articles (**Figure 3C**). The United States is the core of the cooperation network, and the United States and the United Kingdom have the closest cooperation relationship. It is noteworthy that China, as an emerging scientific research force in this field, has a relatively late publication year for its papers, and the level of cooperation needs to be improved. **Figure 3D** illustrates the strong collaboration between various countries in the field of ICI-colitis.

### Institution analysis

**Figure 4:**
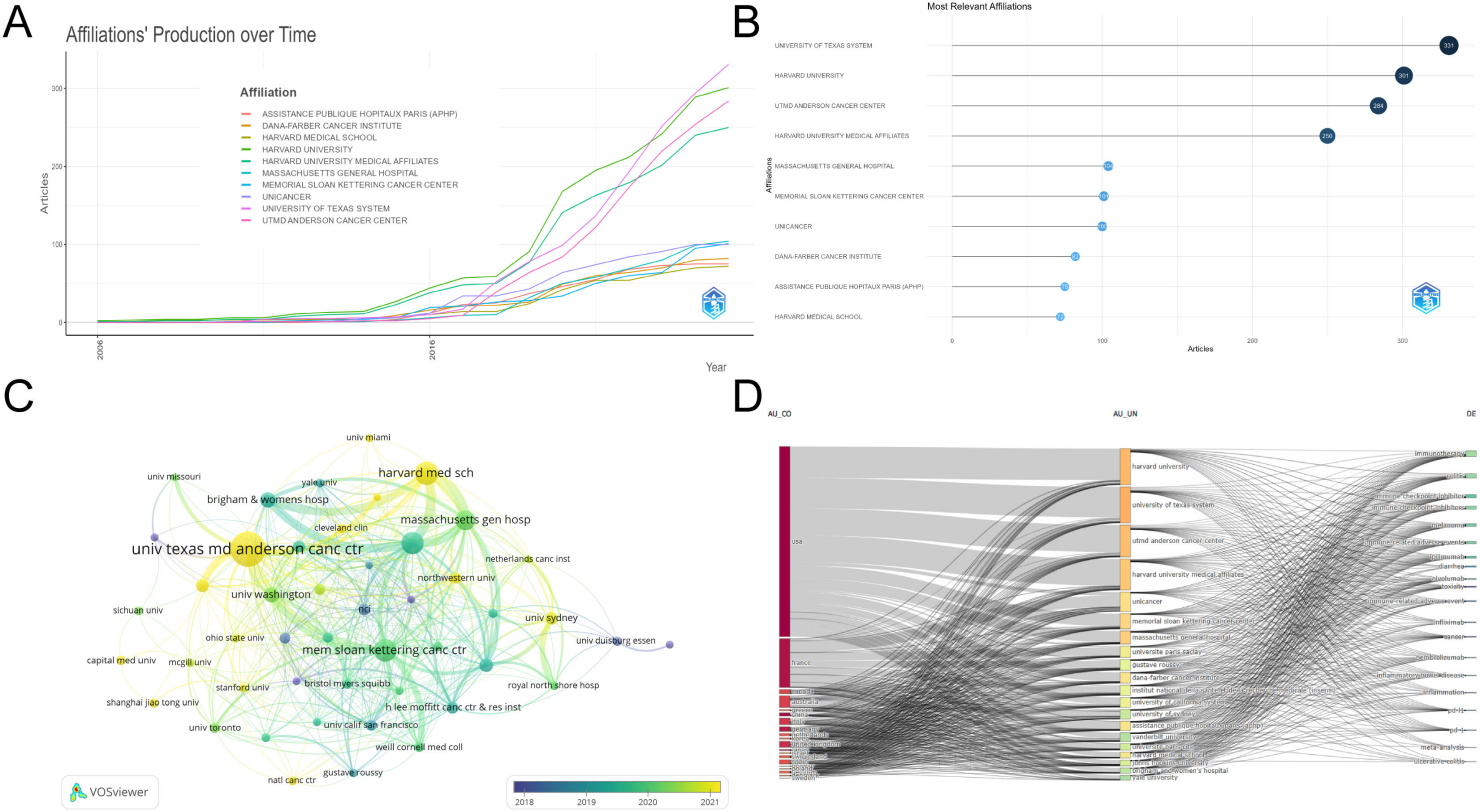
Institutional analysis. **(A)** Top 10 Institutions by Publication Volume Over Time. **(B)** Top 10 Most Connected Institutions. **(C)** Institutional Collaboration Network. **(D)** Tri-domain Map of Countries-Institutions-Keywords.

The present study examined 1,010 papers published by 1,873 institutions. As illustrated in **Table 2**, the top 15 institutions by publication volume are shown. The top three institutions by publication volume were The University of Texas MD Anderson Cancer Center (83), Harvard Medical School (47), and Memorial Sloan Kettering Cancer Center (45). The institutions that received the highest total citations were Memorial Sloan Kettering Cancer Center (11,540), The University of Texas MD Anderson Cancer Center (7,659), and Dana-Farber Cancer Institute (7,287). The Memorial Sloan Kettering Cancer Center exhibited the highest citation count and total connection strength, despite publishing approximately half the number of papers as the University of Texas MD Anderson Cancer Center. In addition, of the 15 institutions/universities with the highest publication volume, 14 are located in the United States, with only one being based in Australia. With respect to the number of articles published by each institution over time (**Figures 4A, B**), these ten institutions/universities have all demonstrated high publication growth rates since 2016, with the University of Texas System and Harvard University exhibiting particularly prominent growth rates. **Figure 4C** illustrates the collaborative networks among institutions. Preliminary analysis indicates that the Memorial Sloan Kettering Cancer Center, Massachusetts General Hospital, and Dana-Farber Cancer Institute have published a higher number of papers in this journal, suggesting that they engage in more collaborative research activities. Subsequently, The University of Texas MD Anderson Cancer Center and Harvard Medical School demonstrated increased publication volume and stronger collaboration capacity and willingness, gradually emerging as rising stars in this bibliometric analysis. Furthermore, to enhance comprehension of the interrelationships among nations, organizations, and keywords, a tri-domain map was employed (**Figure 4D**). Presently, the leading 15 institutions with the highest publication volumes primarily concentrate on immunotherapy, colitis, immune checkpoint inhibitors, and melanoma.

**Table 2:** Top 15 institutions in terms of number of publications.

Rank	Organization	Country	Documents	Citations	Total link strength
1	The University of Texas MD Anderson Cancer Center	USA	83	7659	135
2	Harvard Medical School	USA	47	2649	100
3	Memorial Sloan Kettering Cancer Center	USA	45	11540	137
4	Dana-Farber Cancer Institute	USA	44	7287	137
5	Massachusetts General Hospital	USA	38	5432	106
6	Brigham and Women’s Hospital	USA	26	2091	61
7	University of Washington	USA	26	3638	78
8	Vanderbilt University	USA	22	5177	85
9	Baylor College of Medicine	USA	20	1474	37
10	The University of Sydney	Australia	20	4587	47
11	H. Lee Moffitt Cancer Center & Research Institute	USA	18	4325	52
12	Johns Hopkins University	USA	18	3492	42
13	National Cancer Institute	USA	17	2901	41
14	Northwestern University	USA	16	2379	45
15	University of California, San Francisco	USA	16	2166	42

### Author analysis

**Figure 5:**
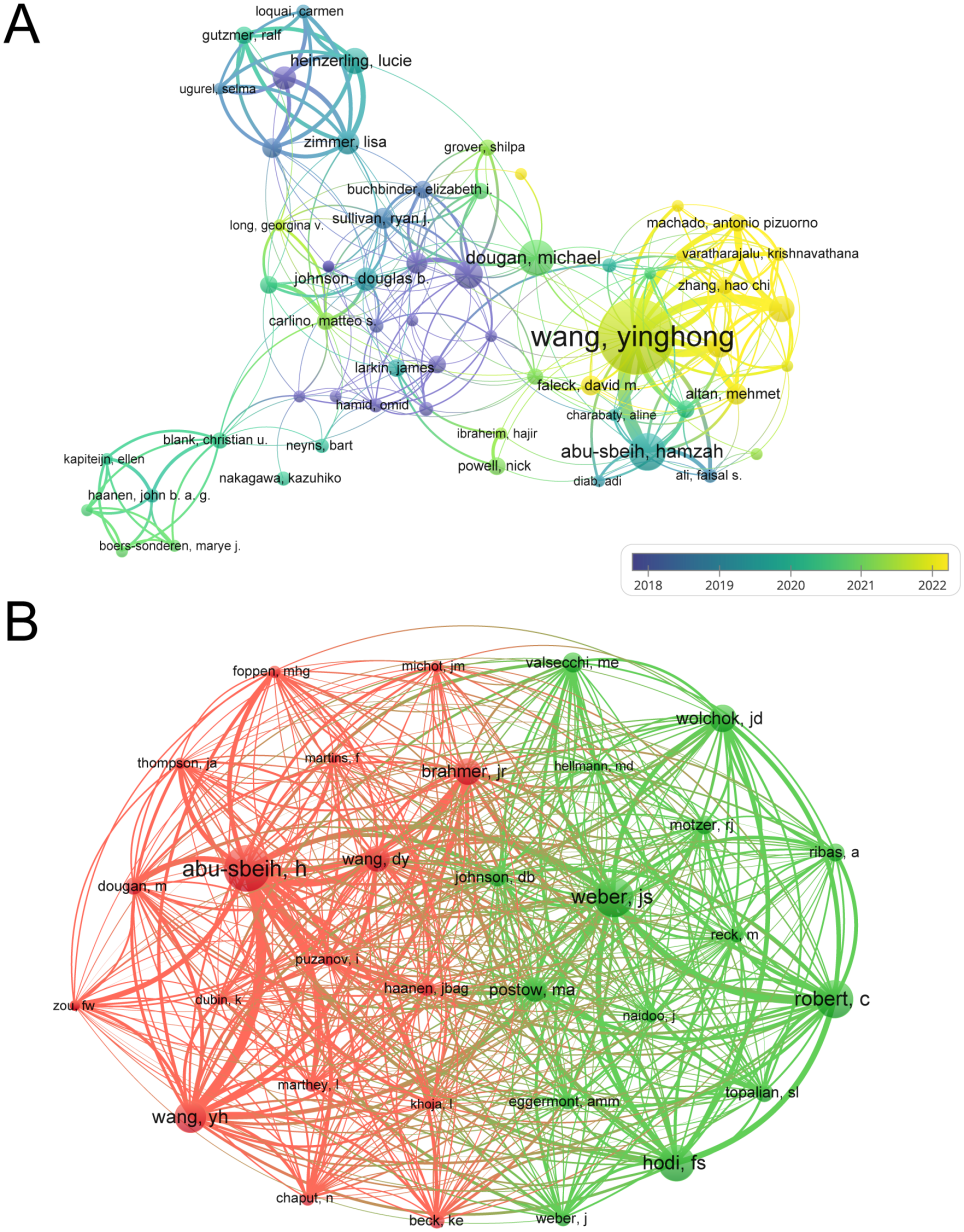
Author co-occurrence analysis related to ICI-induced colitis. **(A)** Author co-occurrence network. **(B)** Author co-citation network.

From 2006 to 2025, a total of 7,385 authors contributed to the publication of papers in this journal, with 61 authors publishing five or more papers. As indicated in **Table 3**, Wang Yinghong has published the highest number of papers (n=48, 4.75%), followed by Abu-Sbeih Hamzah (n=20, 1.98%) and Dougan Michael (n=19, 1.88%). **Figure 5A** presents a collaboration network map of these authors. Historically, scholars such as Abu-Sbeih Hamzah, Hodi, F. Stephen, and Heinzerling, Lucie have played pivotal roles in this field. In recent years, Wang Yinghong, Dougan Michael, and Shatila Malek have emerged as new contributors. Among them, Wang Yinghong is particularly noteworthy for his prolific authorship, having published the highest number of papers in recent years. The co-citation network is illustrated in **Figure 5B**. Co-citation, in this context, denotes the practice of citing multiple authors’ papers within a single article, thereby establishing a co-citation relationship among these authors. Higher co-citation counts are indicative of greater similarity in research outcomes. The co-cited authors can be primarily divided into two clusters. Abu-Sbeih Hamzah et al. (red), and Weber, J.S. et al. (green).

**Table 3:** Top 10 authors and co-cited authors associated with ICI-induced colitis.

Rank	Author	Documents	Citations	Co-cited author	Citations
1	Wang Yinghong	48	2545	Abu-sbeih H	462
2	Abu-Sbeih Hamzah	20	2087	Weber Js	387
3	Dougan Michael	19	1165	Robert C	361
4	Hodi f. Stephen	14	3302	Hodi Fs	342
5	Heinzerling Lucie	13	751	Wang Yh	299
6	Shatila Malek	13	62	Wolchok Jd	254
7	Thomas Anusha s	12	143	Brahmer Jr	246
8	Johnson Douglas b	11	1237	Wang Dy	235
9	Kaehler Katharina c	11	1805	Postow Ma	223
10	Zimmer Lisa	11	711	Valsecchi Me	174

### Distribution of journals

**Figure 6:**
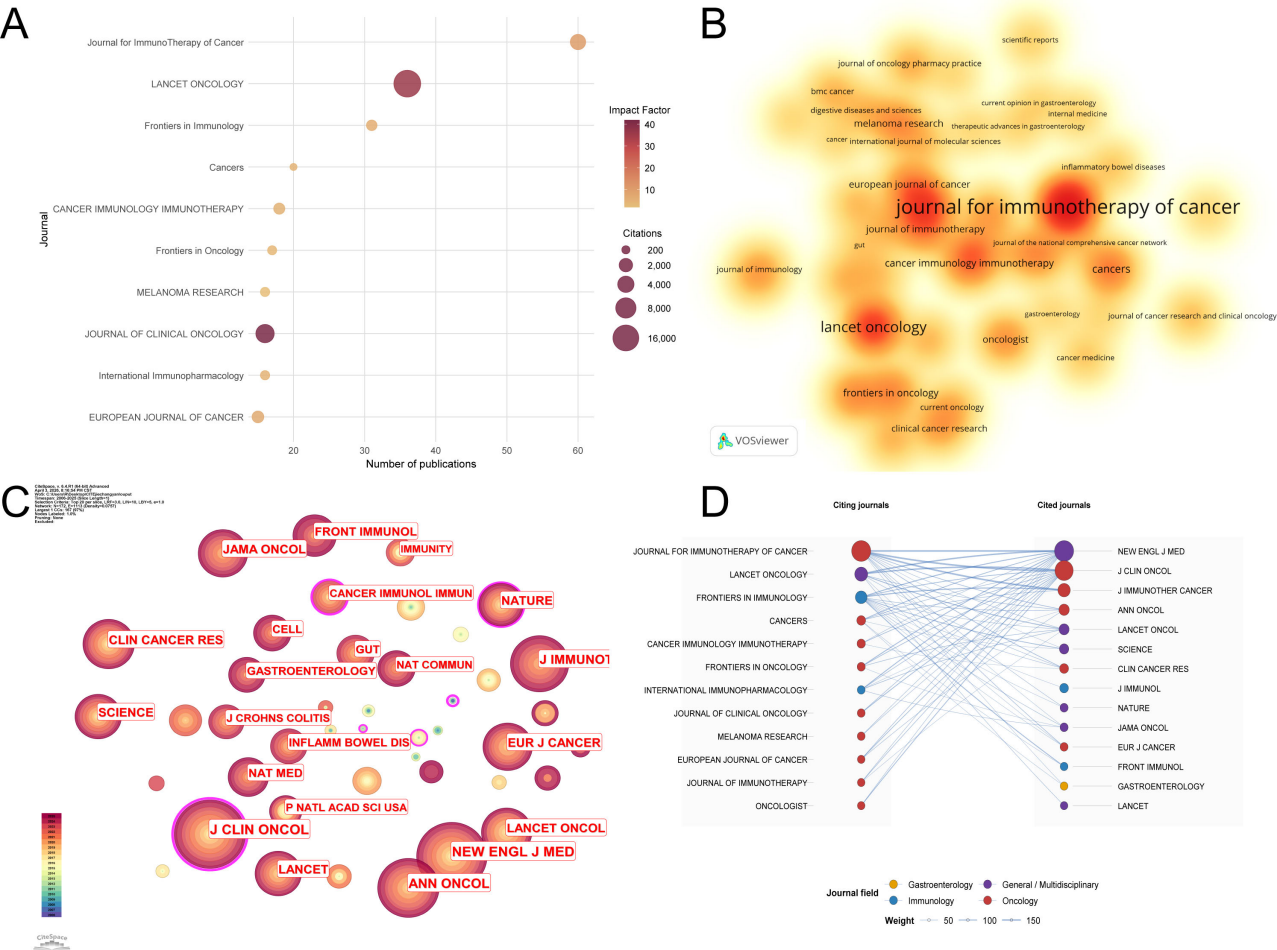
Journal analysis **(A)** Bubble chart of the top 15 journals by publication volume. **(B)** Journal density plot. **(C)** Journal co-citation network map. **(D)** Overlay of journal dual maps for ICI-related colitis.

The analysis of publication source distribution facilitates the identification of high-impact journals within this field. A total of 1,010 papers on ICI-induced colitis were published across 373 journals. **Table 4** presents a comprehensive list of the top 10 journals in this field based on their publication volume. The Lancet Oncology has received a significantly higher number of citations (17,664) compared to other journals in the field. As illustrated in **Figure 6A**, a comprehensive visualization of the publication volume, impact factor, and total citations for the top 10 journals is presented. **Figure 6B** presents a density map of journal publication volumes. Seven of these ten journals are classified in the Q1 quartile, indicating their superior academic standing.

**Table 4:** The top 10 journals that contributed to publications in the field of ICI induced colitis.

Rank	Source	Documents	Percentage (1010)	IF (JCR2024)	Quartile in category	Citations
1	Journal for ImmunoTherapy of Cancer	60	5.94	10.6	Q1	3222
2	LANCET ONCOLOGY	36	3.56	35.9	Q1	17664
3	Frontiers in Immunology	31	3.07	5.9	Q1	719
4	Cancers	20	1.98	4.4	Q2	147
5	CANCER IMMUNOLOGY IMMUNOTHERAPY	18	1.78	5.1	Q1	910
6	Frontiers in Oncology	17	1.68	3.3	Q2	343
7	International Immunopharmacology	16	1.58	4.7	Q1	377
8	JOURNAL OF CLINICAL ONCOLOGY	16	1.58	41.9	Q1	5867
9	MELANOMA RESEARCH	16	1.58	1.9	Q3	378
10	EUROPEAN JOURNAL OF CANCER	15	1.49	7.1	Q1	1160

The influence of a journal is determined by its frequency of co-citations, indicating whether it exerts a significant impact on the scientific community. An analysis of **Figure 6C**, **Table 5** indicates that NEW ENGLAND JOURNAL OF MEDICINE (2710) received the highest number of co-citations, followed by JOURNAL OF CLINICAL ONCOLOGY (2549) and Journal for ImmunoTherapy of Cancer (1450). A study of the top 10 most frequently cited journals reveals that the NEW ENGLAND JOURNAL OF MEDICINE has achieved two notable distinctions. Firstly, it has the highest citation count, and secondly, it has the highest IF (78.5) among these journals. A significant proportion of these ten journals, nine to be precise, have been classified within the top quartile in terms of their journal ranking.

**Table 5:** The top 10 co-cited journals associated with ICI induced colitis.

Rank	Cited journal	Co-citation	IF (JCR2024)	Quartile in category
1	THE NEW ENGLAND JOURNAL OF MEDICINE	2710	78.5	Q1
2	JOURNAL OF CLINICAL ONCOLOGY	2549	41.9	Q1
3	Journal for ImmunoTherapy of Cancer	1450	10.6	Q1
4	ANNALS OF ONCOLOGY	1088	65.4	Q1
5	LANCET ONCOLOGY	1035	35.9	Q1
6	SCIENCE	896	45.8	Q1
7	CLINICAL CANCER RESEARCH	811	10.2	Q1
8	JOURNAL OF IMMUNOLOGY	773	3.4	Q2
9	JOURNAL OF EXPERIMENTAL MEDICINE	647	10.6	Q1
10	NATURE	647	48.5	Q1

The journal bicluster overlay provides a visual representation of the citation relationships among journals across a diverse array of subject areas. This representation serves to elucidate the distribution of prominent journals within research domains and their respective citation networks. **Figure 6D** illustrates the journal-level knowledge flow structure of this research field. The citing journals on the left represent the source journals in which the included studies were published, whereas the cited journals on the right represent the journals most frequently cited in the reference lists of those studies. The links between the two sides indicate aggregated citation relationships between source journals and cited journals; thicker links denote stronger citation pathways, and larger nodes indicate a higher frequency of occurrence of a given journal within the network. Accordingly, this figure provides a concise overview of where the research in this field is primarily published and on which journal-based knowledge foundations it mainly relies, thereby reflecting the core sources of knowledge and the major pathways of cross-journal knowledge transmission.

### Co-cited references and reference bursts

**Figure 7:**
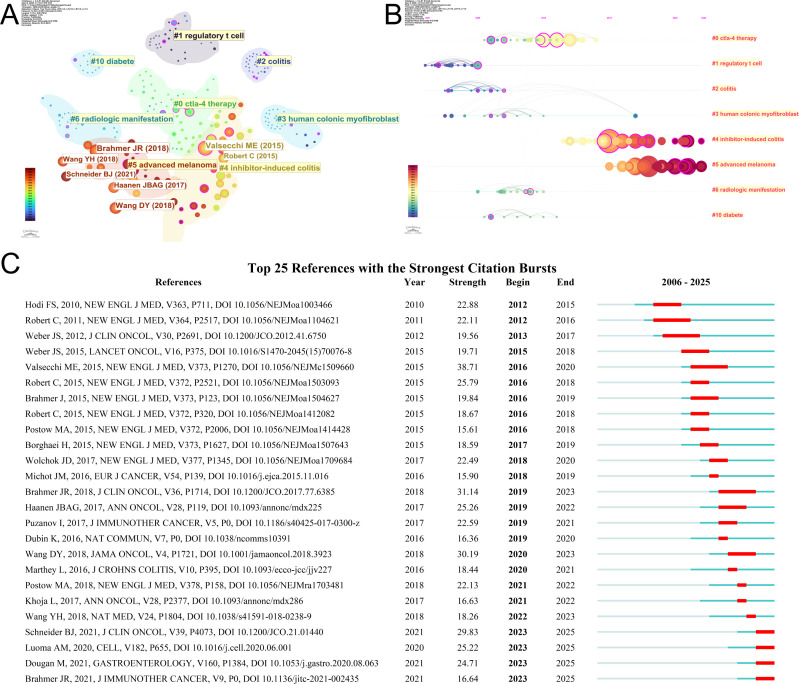
Reference analysis **(A)** Co-cited reference clusters. **(B)** Timeline diagram of co-cited reference clusters. **(C)** Top 25 references with the strongest citation bursts.

A comprehensive analysis of the extant literature reveals a total of 23,430 co-cited references within the domain of ICI-induced colitis. **Table 6** presents the top 10 most frequently cited references on ICI-induced colitis. Among these, the most frequently cited reference (n=208) was a phase III clinical trial spanning 13 countries published by Dr. Hodi et al. in THE NEW ENGLAND JOURNAL OF MEDICINE in 2010. The results of the trial demonstrated that ipilimumab and its combination therapies resulted in survival benefits for patients with metastatic melanoma who had previously received treatment. However, these benefits may be accompanied by adverse events of varying severity. The majority of adverse events demonstrated improvement following the administration of appropriate treatment. A subsequent CiteSpace analysis of reference citations identified eight clusters. The results of the clustering analysis (Q = 0.8412, S = 0.9758) demonstrated analytical reliability (**Figure 7A**). These clusters mainly included topics such as ICI-related colitis and advanced melanoma. The reference timeline analysis shown in **Figure 7B** indicates that early studies primarily focused on mechanism-related topics such as regulatory T cells, after which the research emphasis gradually shifted toward CTLA-4 therapy. In recent years, research attention has tended to concentrate on ICI-related colitis and advanced melanoma. In the current medical literature, inhibitor-induced colitis is a subject of increasing prominence. References accompanied by citation bursts are indicative of cases in which there has been a substantial and abrupt surge in citations over a given interval. We identified the 25 references with the highest citation burst rates (**Figure 7C**), arranged according to the commencement time of their citation bursts. The reference with the strongest citation burst (strength = 38.71) was “Combined Nivolumab and Ipilimumab or Monotherapy in Untreated Melanoma,” authored by James Larkin et al. and published in THE NEW ENGLAND JOURNAL OF MEDICINE, with a citation burst period from 2016 to 2020. In addition, recently emerging burst references have mainly focused on clinical management guidelines and therapeutic strategies such as fecal microbiota transplantation (FMT). Overall, **Figure 7** highlights an evolution in the knowledge structure of this field, from early mechanistic investigations to a growing emphasis on the clinical management and treatment of ICI-related colitis, reflecting its increasing translational potential.

**Table 6:** Top 10 co-cited references of ICI induced colitis.

Rank	Journal	Citations	Year	Type	Doi
1	THE NEW ENGLAND JOURNAL OF MEDICINE	208	2010	clinical trial	10.1056/NEJMoa1003466
2	THE NEW ENGLAND JOURNAL OF MEDICINE	174	2015	clinical trial	10.1056/NEJMoa1504030
3	Journal of Clinical Oncology	133	2018	Practice Guideline	10.1200/JCO.2017.77.6385
4	JAMA Oncology	126	2018	Meta-Analysis	10.1001/jamaoncol.2018.3923
5	Journal of Clinical Oncology	125	2006	Retrospective Cohort Analysis	10.1200/JCO.2005.04.5716
6	THE NEW ENGLAND JOURNAL OF MEDICINE	110	2018	Review	10.1056/NEJMra1703481
7	THE NEW ENGLAND JOURNAL OF MEDICINE	106	2015	clinical trial	10.1056/NEJMoa1503093
8	Journal of Clinical Oncology	104	2012	Review	10.1200/JCO.2012.41.6750
9	ANNALS OF ONCOLOGY	99	2017	Practice Guideline	10.1093/annonc/mdx225
10	Journal of Crohns & Colitis	99	2016	Retrospective Cohort Analysis	10.1093/ecco-jcc/jjv227

### High-impact articles

**Table 7** presents a comprehensive list of the top 10 most cited articles on ICI-induced colitis. The most frequently cited article is “Phase I study of single-agent anti-programmed death-1 (MDX-1106) in refractory solid tumors: safety, clinical activity, pharmacodynamics, and immunologic correlates” by Brahmer Jr., published in the Journal of Clinical Oncology in 2010, with 2,485 citations. The present document thoroughly delineates a Phase I clinical trial that was conducted for the purpose of evaluating the efficacy of anti-PD-1 (MDX-1106) therapy in the treatment of refractory solid tumors. The aforementioned tumors include advanced metastatic melanoma, colorectal cancer, castration-resistant prostate cancer, non-small cell lung cancer, and renal cell carcinoma. The study evaluated safety, tolerability, and pharmacodynamic parameters, establishing a foundation for subsequent immune checkpoint inhibitor therapies in cancer treatment.

**Table 7:** Top 10 high-impact articles on ICI induced colitis.

Author	Journal	IF	Publication year	Total citations	TC per year	DOI
BRAHMER JR	JOURNAL OF CLINICAL ONCOLOGY	41.9	2010	2485	155.31	10.1200/JCO.2009.26.7609
WANG DY	JAMA Oncology	20.1	2018	1898	237.25	10.1001/jamaoncol.2018.3923
MARABELLE A	LANCET ONCOLOGY	35.9	2020	1712	285.33	10.1016/S1470-2045(20)30445-9
RIBAS A	LANCET ONCOLOGY	35.9	2015	1280	116.36	10.1016/S1470-2045(15)00083-2
KWON ED	LANCET ONCOLOGY	35.9	2014	1231	102.58	10.1016/S1470-2045(14)70189-5
WEBER JS	JOURNAL OF CLINICAL ONCOLOGY	41.9	2012	1145	81.79	10.1200/JCO.2012.41.6750
HODI FS	LANCET ONCOLOGY	35.9	2018	1078	134.75	10.1016/S1470-2045(18)30700-9
BALAR AV	LANCET ONCOLOGY	35.9	2017	1030	114.44	10.1016/S1470-2045(17)30616-2
EGGERMONT AMM	LANCET ONCOLOGY	35.9	2015	995	90.45	10.1016/S1470-2045(15)70122-1
ROYAL RE	JOURNAL OF IMMUNOTHERAPY	2.9	2010	971	60.69	10.1097/CJI.0b013e3181eec14c

### Keyword analysis

**Figure 8:**
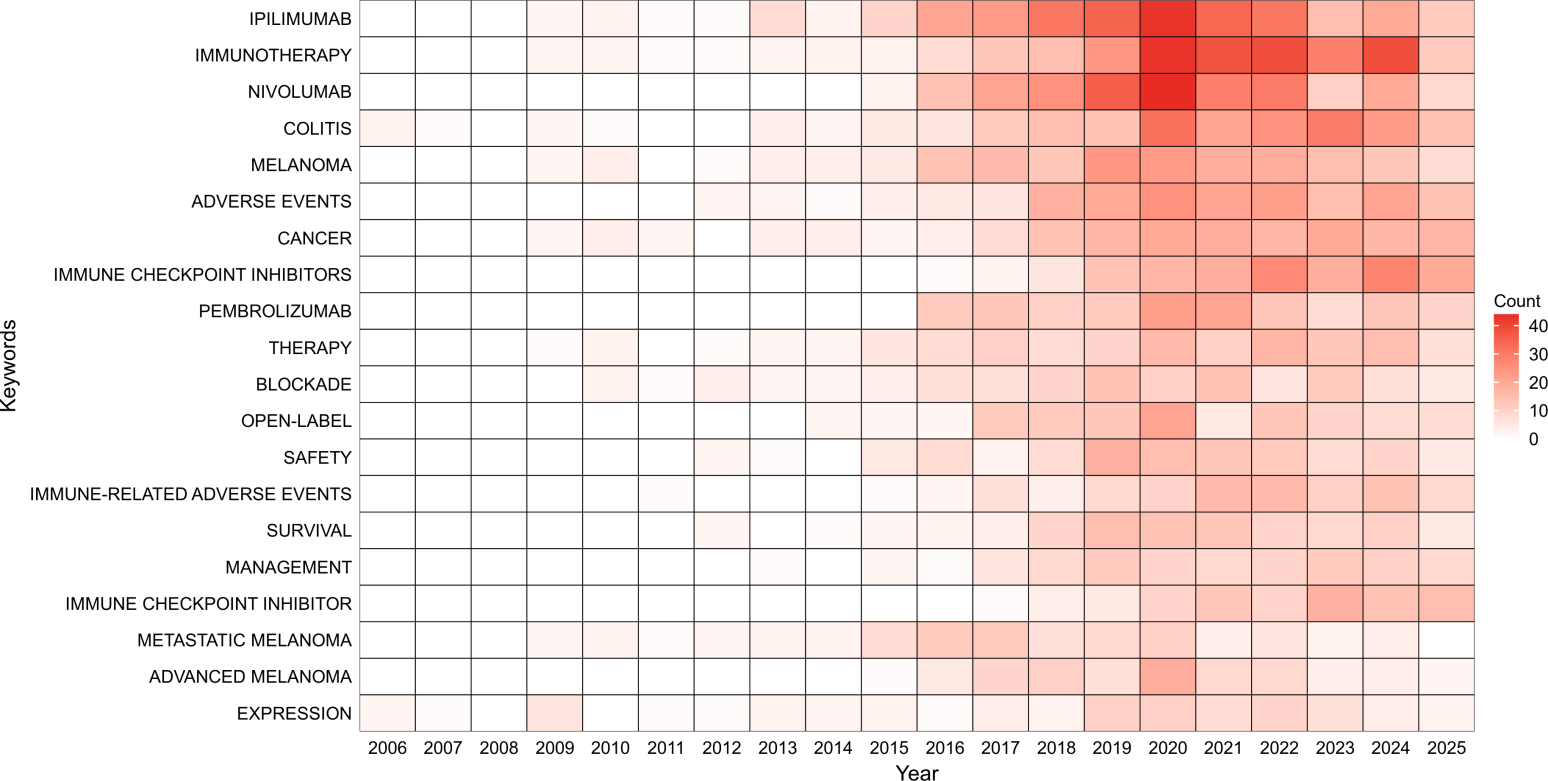
Annual heatmap of keywords. Annual keyword popularity is measured by annual keyword frequency.

**Figure 9:**
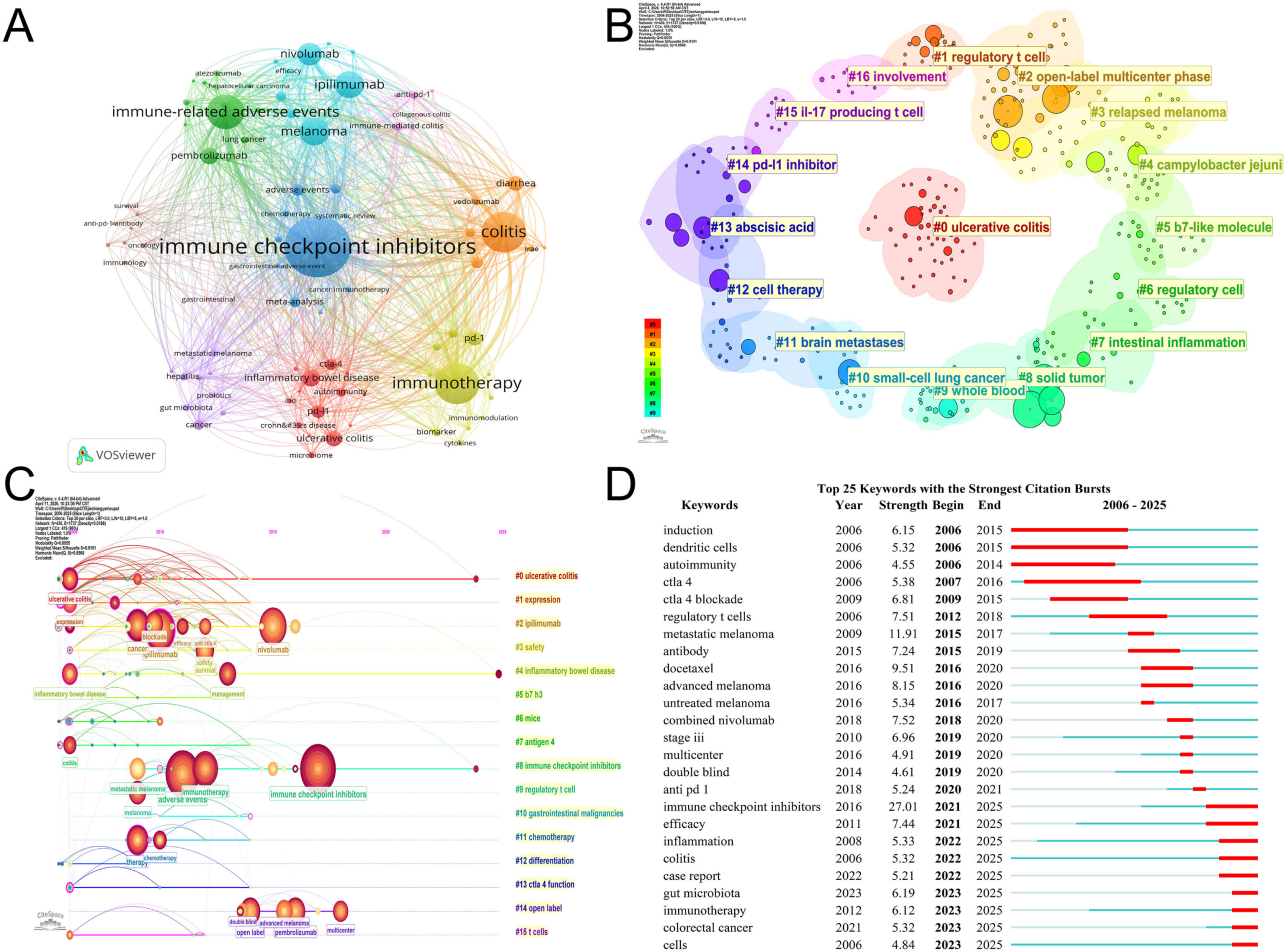
Keyword analysis. **(A)** Keyword co-occurrence map. **(B)** Keyword clusters. **(C)** Keyword timeline. **(D)** Top 25 keywords with highest citation burst rates.

By analyzing keywords, one can swiftly comprehend the nuances of a given field and its evolving trends. **Figure 8** presents an annual heatmap of keywords associated with ICI-induced colitis from 2006 to 2025, measured by the number of keyword occurrences per year. The co-occurrence of keywords in this field was analyzed based on the frequency of their joint appearance in authors’ papers (**Figure 9A**). Beyond immune checkpoint inhibitors and colitis, the most frequently occurring keywords were immunotherapy (n=169), irAE (n=134), melanoma (n=97), ipilimumab (n=94), and nivolumab (n=73). As illustrated in **Figure 9B**, the results of the clustering process are displayed. The clustering results (Q = 0.8055, S = 0.9151) underscore the reliability of the findings. The results of the analysis indicate that the keywords were primarily grouped into 17 categories. **Figure 9C** presents the keyword timeline diagram constructed for the present study. The visual depiction of the research hotspots and future development directions within the journal’s studies from a temporal perspective facilitates the identification of the evolutionary trajectory and stage characteristics of the research field. **Figure 9D** presents a visual representation of the top 25 keywords that have exhibited the most significant citation bursts. Among these, immune checkpoint inhibitors, metastatic melanoma, and docetaxel exhibit the highest burst intensity. It is evident that immune checkpoint inhibitors demonstrate the most pronounced burst intensity. The emergence of this keyword occurred in 2016, and it underwent a significant increase in usage in 2021. Notably, in recent years, keywords such as “gut microbiota,” “immunotherapy,” “colorectal cancer,” and “case report” have shown marked citation bursts, which is consistent with the current research trends and major areas of focus in this field.

### Changes in research trends

**Figure 10:**
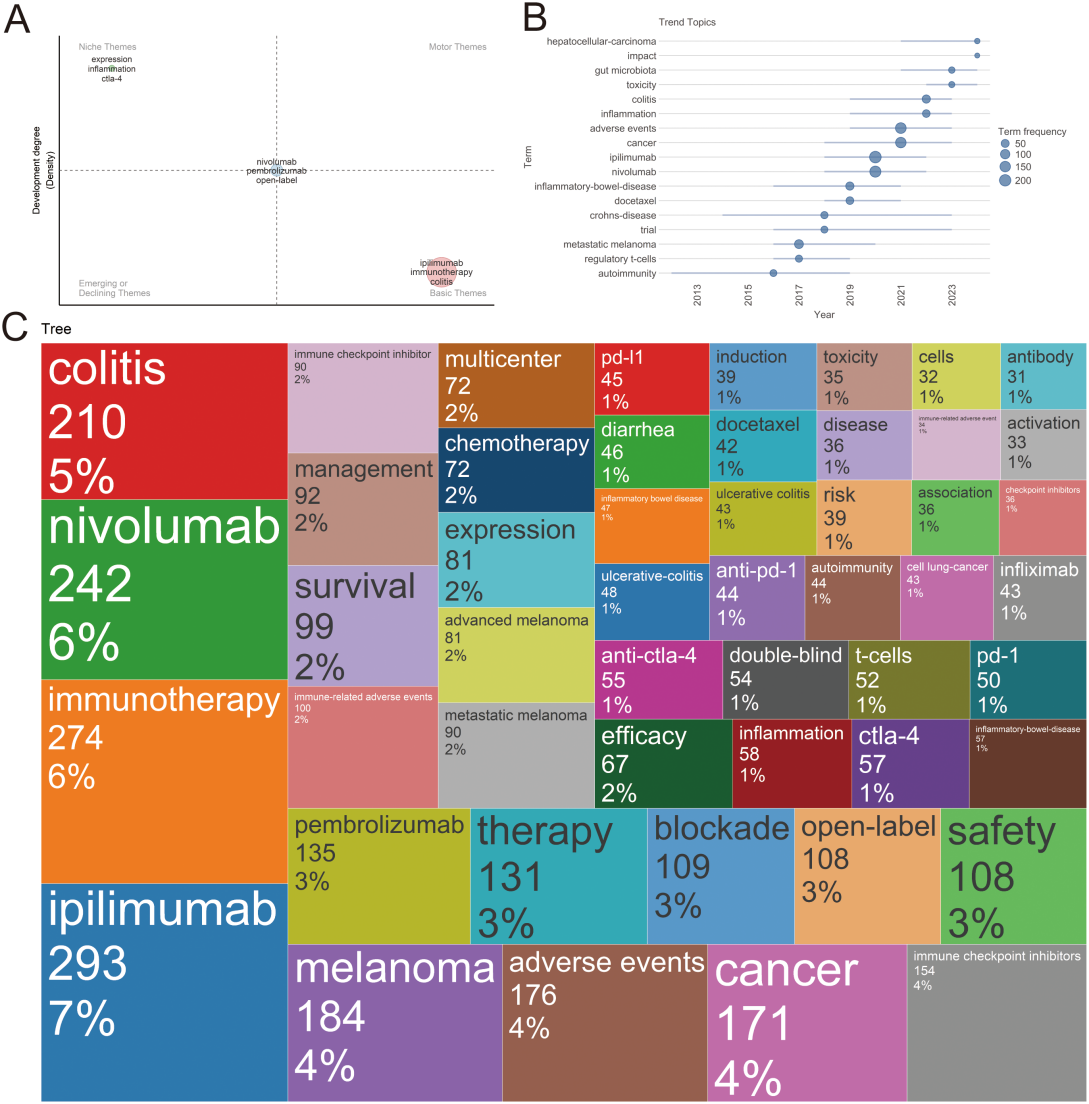
Thematic trend analysis. **(A)** Thematic Evolution Map. **(B)** Thematic Trend Chart. **(C)** Dendrogram.

Thematic term analysis was employed to explore shifts in publication trends within the field of ICI-induced colitis (**Figure 10A**). A preliminary investigation of themes identified in ICI-colitis reveals the presence of three basic themes: ipilimumab, immunotherapy and colitis. It is noteworthy that the themes nivolumab, pembrolizumab, open-label occupied central positions in the thematic map, exhibiting intermediate levels of centrality and density. The thematic trend map (**Figure 10B**) suggests that hepatocellular carcinoma and gut microbiota may represent potential future development trends. **Figure 10C** provides a more intuitive visualization of keyword distribution, consistent with our previously observed findings.

## Discussion

### General information

This study presents a relatively recent and comprehensive bibliometric analysis of publications on ICI-mediated colitis from 2006 to 2025. Ipilimumab was the first CTLA-4 antibody approved by the U.S. Food and Drug Administration (FDA) in 2011 for treating advanced (metastatic) melanoma (17). Subsequently, in 2014, the FDA approved the first PD-1/PD-L1 inhibitors, nivolumab and pembrolizumab, for treating certain cancer types (18). To date, multiple types of ICIs have been approved for various cancers (19). With the widespread adoption of ICIs in oncology, case reports of irAEs have increased, drawing greater attention to their adverse effects on patients. The 2018 Nobel Prize in Physiology or Medicine was jointly awarded to James P. Allison and Tasuku Honjo for their discovery of cancer therapy through the inhibition of negative immune regulation. This further fueled research enthusiasm in the field. From January 1, 2025, to August 7, 2025, 72 articles have been published in this field, maintaining a high level of activity. We believe research in this field will continue to attract widespread attention and remain a prominent trend in tumor immunotherapy.

In terms of publication output, the United States ranked first, followed by China and Japan. The United States also showed the highest total citation count and strongest collaboration links, indicating its central position in this research network. China ranked second in publication volume, but its citation-based indicators were lower than those of several Western countries. Because bibliometric indicators are influenced by publication timing, collaboration patterns, journal distribution, and citation accumulation windows, these differences should be interpreted cautiously. In the present dataset, Chinese publications entered the field relatively later and showed lower international collaboration intensity than the United States and several European countries, which may partly contribute to lower citation visibility.

Regarding collaboration, we observe that the United States maintains close partnerships with countries such as the United Kingdom, France, Germany, Australia, China, and Canada. Historically, collaborative efforts were centered around the United States, but in recent years, the focus has gradually shifted toward China and Japan. Despite this, China possesses a large pool of clinical patients, making it a valuable source for multicenter clinical trials. Through enhanced collaboration with other nations and increased financial investment, Chinese researchers could potentially produce higher-quality studies, thereby advancing the development of cancer immunotherapy.

The top 10 most cited publications in this study were published between 2010 and 2020, focusing on clinical trials of Ipilimumab for various solid tumors and discussions of ICI-induced toxicities. We also analyzed keywords in this domain. Among them, immunotherapy, irAE, ipilimumab, Pembrolizumab, and nivolumab were the most frequently occurring keywords beyond ICI and colitis, indicating they have become key research focal points in this field. Taken together, these patterns suggest that the field is no longer developing as a purely descriptive toxicity literature, but is increasingly moving toward clinically structured and mechanistically informed investigation.

### Comparison with other organ-specific irAE bibliometric analyses

Although, to the best of our knowledge, the present study is the first bibliometric analysis specifically focused on ICI-mediated colitis, its novelty should not be understood merely in chronological terms. More importantly, the distinctive value of this work lies in the fact that, compared with bibliometric mappings of other organ-specific irAEs, including pneumonitis, rheumatic irAEs, and endocrine toxicities, colitis occupies a substantially different position in the translational research landscape. Previous bibliometric studies in adjacent irAE fields have largely emphasized epidemiology, risk stratification, radiologic diagnosis, and endocrine replacement strategies (20–22). By contrast, the ICI-colitis literature is more strongly centered on immunopathology, biomarker discovery, and microbiota-directed therapeutic approaches. In other words, colitis is not simply another organ-specific toxicity to be mapped, but rather one of the few irAEs that has already begun to reshape therapeutic decision-making.

The knowledge structure identified in our analysis supports this distinction. First, both the keywords co-occurrence and keywords citation burst in the colitis field increasingly converge on topics such as fecal microbiota transplantation, gut microbiota, and inflammation, indicating a gradual shift toward intervention-oriented and biologically stratified investigation. Second, compared with pneumonitis or endocrine irAEs, ICI-colitis has already accumulated a larger body of literature related to steroid-refractory disease, endoscopic assessment, and microbiota remodeling (23–25). This suggests that a colitis-centered knowledge network is progressively taking shape, one that is primarily organized around gastroenterology, immune pathology, and refractory disease management. These features make bibliometric analysis in ICI-colitis more directly relevant to clinical practice.

This distinction becomes even more meaningful when considered in light of current practice guidelines. Both ASCO and NCCN recommend that immune-mediated diarrhea/colitis should be regarded as a toxicity requiring intensified clinical attention, including graded evaluation, corticosteroid therapy, and escalation to selective immunosuppressive agents such as infliximab or vedolizumab in refractory cases (23, 25). Accordingly, in our analysis, when citation bursts converge on Wang yinghong, Dougan M, and FMT-related studies, these signals should not be interpreted merely as bibliometric hotspots. Rather, they should be understood as markers of unresolved clinical needs: how to identify refractory patients earlier, how to control intestinal inflammation while preserving antitumor efficacy, and how to reduce recurrent or prolonged exposure to systemic immunosuppression. It is precisely at this point that the bibliometric profile of colitis differs from that of many other irAEs.

Notably, the rise of microbiota-centered research further reinforces the particular importance of the colitis field. Early studies demonstrated that FMT could rescue refractory ICI-related colitis and was accompanied by reconstruction of the gut microbiota and increased Treg-associated features in the colonic mucosa (26). More recent translational studies have further suggested that FMT not only induces remission in steroid-refractory disease but also remodels microbial diversity and mucosal immune composition (24). At the same time, microbiome-related studies suggest that taxa such as *Akkermansia muciniphila* may have predictive value for immunotherapy response (27). With regard to biomarker development for colitis itself, fecal calprotectin has been shown to be useful for assessing endoscopic and histologic remission in immune-mediated colitis (28), while anti-integrin αvβ6 autoantibodies have been proposed as a potential biomarker for ulcerative colitis-like ICI-related colitis (29). Collectively, these findings place ICI-colitis at the intersection of toxicity biology, biomarker discovery, and host–microbiome therapeutic strategies. Such characteristics have not been similarly highlighted in bibliometric studies of organ-specific irAEs such as pneumonitis or endocrine toxicities. For this reason, ICI-colitis should be regarded as a clinically meaningful research domain rather than “yet another organ-specific map”.

Taken together, the principal contribution of the present study is not simply that it represents the first bibliometric report dedicated to ICI-related colitis, but that it reveals how this field has evolved from early toxicity recognition in the PD-1/CTLA-4 era, to guideline-driven clinical management, and more recently to microbiota-centered precision intervention. This evolutionary trajectory carries greater translational significance than the framework presented in many bibliometric studies of other organ-specific irAEs.

### Mechanistic insights from keyword and reference clusters

An important implication emerging from our bibliometric findings is that the field of ICI-colitis has evolved along a trajectory that is increasingly amenable to mechanistic interpretation. Specifically, the reference timeline and citation burst analyses suggest that the early stage of this field was largely dominated by landmark anti-CTLA-4 and anti-PD-1 clinical trials, followed by a second phase centered on toxicity recognition and guideline-oriented management. More recently, the field has entered a translational stage characterized by growing attention to mucosal immunology, epithelial barrier dysfunction, microbiota remodeling, and biomarker development. Our results show that the earliest highly cited publications were mainly concentrated on melanoma trials from the ipilimumab era and toxicity reports, whereas more recent keyword bursts have shifted toward inflammation, gut microbiota, and biomarker-related themes. This transition indicates that the field has moved beyond describing clinical incidence and severity toward understanding pathogenic mechanisms and identifying potentially actionable targets.

From a mechanistic perspective, research on ICI-related colitis appears to be progressively converging on three interconnected directions. The first is excessive T-cell activation, particularly within the intestinal mucosa, as reflected by regulatory T-cell imbalance, the transition of CD8 tissue-resident memory T cells toward cytotoxic effector states, and the breakdown of mucosal immune tolerance (30). The second is cytokine-mediated inflammatory amplification. Pathways involving IL-6, IL-17, IL-23, and TNF not only contribute to the persistence of intestinal inflammation, but also suggest that this process is not merely an epiphenomenon of immune activation, but rather a selectively targetable therapeutic axis (31). Third, dysregulation of the gut microbiota and epithelial barrier has become a major translational focus, with increasing attention shifting from descriptive microbial profiling toward microbiota remodeling and mucosal immune reconstitution in refractory colitis (26). Parallel mechanistic studies have further highlighted epithelial barrier dysfunction and epithelial–immune crosstalk as key components of disease pathogenesis (32). In this context, FMT-related studies have played an especially important role in advancing microbiota-directed intervention in refractory disease (24).

Against this background, biomarker research has also begun to move away from conventional immunotherapy predictive markers toward monitoring and stratification tools that are more directly relevant to ICI-colitis itself. Fecal calprotectin represents a noninvasive inflammatory monitoring approach and may be useful for assessing endoscopic and histologic activity (29). Anti-αvβ6 is more closely aligned with a colitis-specific biomarker framework and has been associated with ulcerative colitis-like phenotypes, severe colitis, and steroid resistance (28). Meanwhile, gut microbiota-related features suggest that future biomarker strategies may no longer rely on single-molecule indicators alone, but instead increasingly incorporate host–microbiota interaction patterns to assess disease susceptibility, therapeutic response, and prognosis (33).

It should also be emphasized that ICI-related colitis is not a biologically uniform field. Gastrointestinal toxicities differ substantially according to the class of immune checkpoint inhibitor. Anti-CTLA-4 therapy is generally associated with earlier onset and more severe colitis, whereas PD-1/PD-L1 inhibitor-associated colitis tends to be relatively milder, while combination therapy usually confers the highest gastrointestinal toxicity burden (34, 35). In addition, the current literature remains heavily dominated by melanoma cohorts, and the incidence patterns and clinical manifestations of gastrointestinal irAEs are not entirely consistent across different tumor types (36). At the same time, microbiome-related studies suggest that baseline microbial composition may influence not only susceptibility to colitis, but also antitumor efficacy (27). Taken together, these stratification features indicate that the bibliometric landscape of ICI-colitis should not be interpreted as a homogeneous body of literature, but rather understood within a framework shaped by drug class, tumor context, and host microbiota heterogeneity.

Overall, the keyword bursts, reference timeline, and thematic evolution collectively support a three-stage interpretation of the field: (1) an early toxicity-recognition stage during the ipilimumab/early immune checkpoint inhibitor era; (2) a guideline-development and steroid-refractory management stage; and (3) the current translational stage oriented toward microbiota, barrier biology, and biomarker development.

### Mechanism of ICI induced colitis

#### Overview

**Figure 11:**
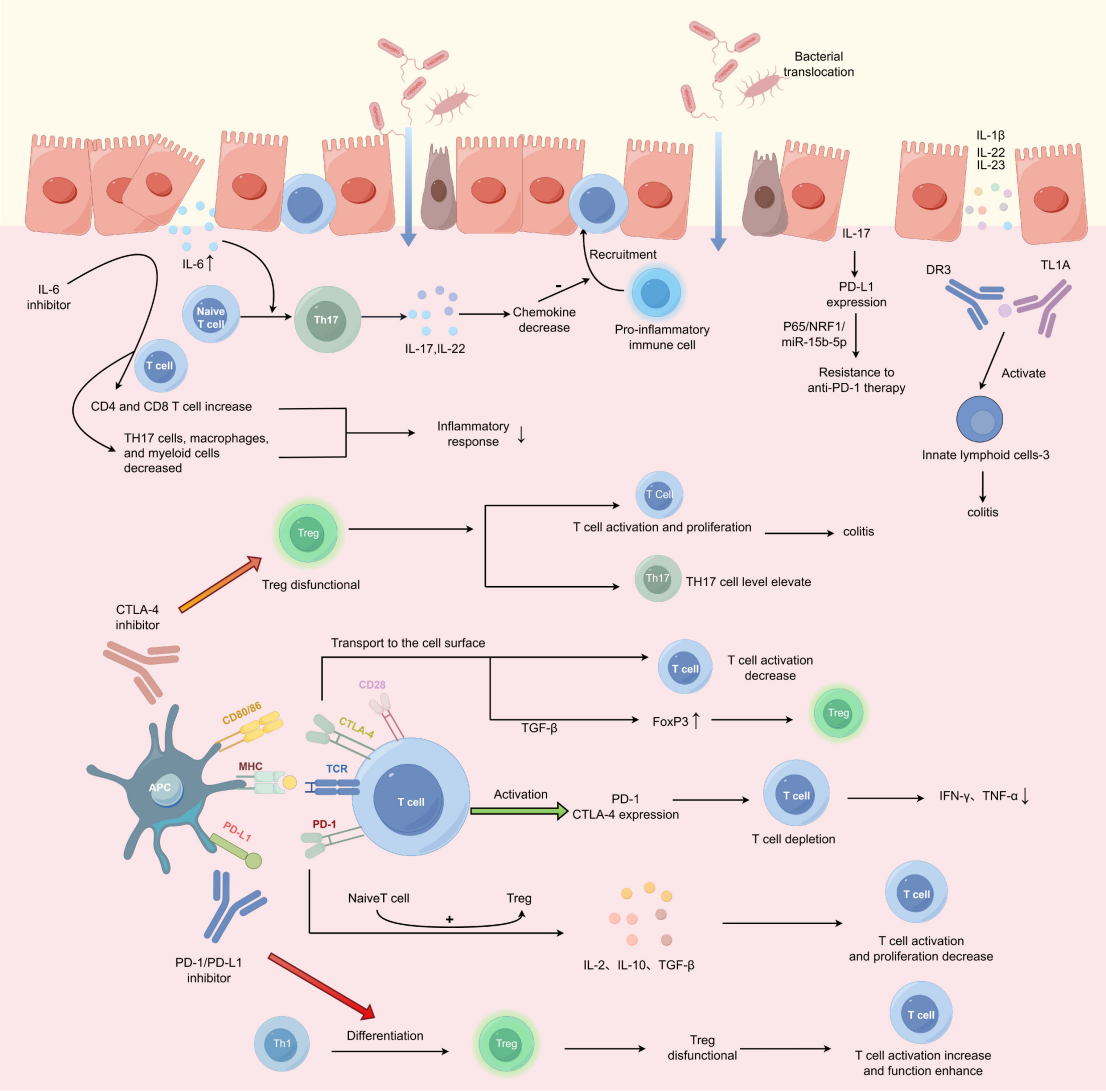
Mechanism of ICI-mediated colitis. (1) Under physiological conditions, antigen presentation by APCs via MHC to the TCR activates T cells, with a co-stimulatory signal from CD28 on T cells binding to CD80/86 on APCs. Activated T cells proliferate and upregulate PD-1 and CTLA-4 expression. PD-1 binding to its ligands promotes naïve CD4^+^ T cell differentiation into Tregs, enhancing secretion of immunosuppressive cytokines like IL-2, IL-10, and TGF-β, thereby suppressing T cell function. CTLA-4, upon binding CD80/CD86, translocates to the cell surface, inhibiting T cell activation and promoting Treg generation. Both pathways upregulate Tregs while inducing T cell dysfunction. (2) PD-1 inhibitors block the differentiation of Th1 cells into Tregs, suppress Treg function, and promote T cell activation and enhanced effector function. CTLA-4 inhibitors downregulate Treg function, activate T cells, and drive their proliferation. Additionally, CTLA-4 inhibition upregulates TH17 cell levels, ultimately mediating colitis. (3) IL-6 regulates naïve T cell differentiation into TH17 cells, which produce cytokines such as IL-17 and IL-22. These cytokines can suppress chemokine expression and block the infiltration of pro-inflammatory immune cells into the colon, conferring resistance to colitis and colon cancer. Other cytokines, including IL-1β, IL-22, IL-23, and TL1A, are also involved in the pathophysiology of colitis. The gut microbiota may play a dual regulatory role in the initiation and progression of colitis. Created by Figdraw (RIWPO5dd53).

Consistent with the keyword bursts and reference-cluster evolution observed in our scientometric analysis, the mechanistic literature on ICI-colitis has increasingly converged on three interrelated processes: aberrant T-cell activation, cytokine-amplified mucosal inflammation, and gut microbiota/barrier dysregulation. At present, the potential mechanism of ICI induced colitis is still unclear. However, extensive research has shown that ICI induced colitis is associated with excessive activation of T cells, cytokine imbalance and intestinal microbial imbalance (37). The following sections will develop discussion along these three dimensions (**Figure 11**).

#### Overactivation of T cells

Generally, two signals are required for T cell activation: APC presents antigens on the major histocompatibility complex MHC and binds to T cell receptors on the surface of CD4/8T cells; secondly, a synergistic stimulatory signal generated by the binding of CD80 (B7-1) and CD86 (B7-2) on the surface of APC with CD28 on T cells (38, 39). T cell activation leads to their proliferation, production of cytokines, and expression of CTLA-4 and PD-1 (40). ICI exerts an interfering effect by affecting different stages of T cell activation. ICI plays a role by removing the braking effect of the immune system, mainly including naïve T cell activation and reactivation of memory antitumor T cell responses (41). However, some scholars believe that ICI mainly restores the normal function of the immune system rather than enhances its function (42).

PD-1 is mainly expressed on activated T cells, B cells, natural killer T cells, activated monocytes and dendritic cells (DC) (43, 44). Continued expression of PD-1 on T cells induces T cell depletion, and depleted CD8 +T cells lose their effector functions, manifesting themselves as unable to secrete pro-inflammatory cytokines such as IFN-γ, TNF-α and others (45). Binding of PD-1 to its ligand inhibits TCR and CD28 signaling (46, 47), thereby inhibiting cellular functions such as activation, proliferation, metabolic regulation, cytotoxicity and cytokine production (48). In addition, this binding can also promote the transformation of naive CD4 +T cells into Treg, thereby enhancing the effects of immunosuppressive Treg cells (49). Treg cells can produce immunosuppressive cytokines such as IL-10, IL-2 and TGF-β, thereby inhibiting T cell activation and proliferation (50). PD-1 and PD-L1 inhibitors inhibit the differentiation of Th1 into Treg, resulting in a decrease in Treg survival and immunosuppression functions, thereby enhancing T cell activation and increasing the production of cytokines.

CTLA-4 is primarily expressed by T cells. It resides within intracellular compartments and is scarcely detectable on the cell surface (51). CTLA-4 exhibits higher affinity for CD80 and CD86, competing with CD28 for ligand binding (52). Subsequently, the CTLA-4-CD80/CD86 complex is transported to the cell surface, ultimately inhibiting T cell activation (51). Furthermore, CTLA-4 is implicated in TGF-β-induced upregulation of FoxP3 expression, thereby promoting Treg generation (53). However, CTLA-4 inhibitors induce multiple cellular alterations, including T cell activation and proliferation, impaired regulatory T cell (Treg) survival, and increased levels of TH17 cells. Anti-CTLA-4 therapy can induce Treg exhaustion, leading to excessive activation of effector T cells such as cytotoxic T lymphocytes, resulting in ICI-induced colitis (54).

Gupta et al. used single-cell and spatial transcriptomic analysis to find that ICI binding cells are mainly CD4 T cells, which are enriched in peripheral helper T cells, follicular helper T cells and Treg (55). A high-throughput sequencing study of Merkel cell carcinoma patient tissues indicates that ICI-induced colitis manifests as distinct T cell subsets defined by cytotoxic, memory, and proliferative markers across different stages. Circulating and colon-resident T cell subsets may represent underlying drivers of the inflammatory response (56). Single-cell analysis of immune cell populations in colitis revealed expansion of CTLA-4+ Treg cells in patients, alongside differentiation of CD8 tissue-resident memory T cells (Trm) toward cytotoxic effector cells (57). Sasson et al. and van Eijs et al. identified CD8 tissue-resident memory T cells as the predominant activated T cell subset. The activation level of CD8 T+RM cells positively correlated with the clinical severity of ICI-induced colitis. CD8+TRM cells expressed high levels of checkpoint inhibitors and IFN-γ transcripts in intestinal tissues (30, 58). This suggests that the activation and differentiation of TRM cells in the gastrointestinal tract may be a key driver of colitis. Another recent study similarly observed a predominance of cytotoxic T cells over helper T cells (59). In colitis samples, it was found that CD8+ resident memory T cells and cytotoxic CD8T cells and CD4+ resident memory T cells were significantly enriched. Both of these RMT cells are precursor cells and can differentiate into cytotoxic effector cells (60). Increased colonic CD8+ T cell counts have been reported to correlate with clinical severity and failure of first-line steroid therapy (61). Furthermore, in melanoma patients receiving combined anti-PD-1 and anti-CTLA-4 therapy, two pre-treatment factors in peripheral blood—abundance of activated CD4 memory T cells and TCR diversity—were associated with severe irAEs (62). However, different ICI types determine distinct biological characteristics in colitis patients. In severe colitis following ICI treatment, high CD4+ T cell infiltration is more prevalent in anti-CTLA-4-treated patients, whereas high CD8+ T cell infiltration typically occurs in anti-PD-1-induced colitis; Tregs predominate in PD-1-induced colitis, whereas elevated TNF-α concentrations are observed in CTLA-4-induced colitis (63). In addition, T cells in patients with colitis showed systemic expansion after the initiation of immunotherapy, which mainly consisted of tissue-resident memory CD8T cells and Th1-type CD4 T cells, of which CD8 T cell expansion was locally induced, and Th1 cell expansion had systemic characteristics (64). These findings suggest that T cells of diverse types and developmental stages contribute to the pathogenesis and progression of ICI-induced colitis.

The aforementioned immune imbalance leads to enhanced activation of CD4+ T cells and CD8+ T cells, which in turn causes damage to normal cells. Additionally, ICI inhibitors result in increased production of cytokines such as TNF, IFN-γ, and IL-17. These cytokines not only promote T cell activation and proliferation but also exert pro-inflammatory effects. As mentioned in the study by Bernard C Lo et al, IFN-γ-producing CD4T cells are not activated in a controlled manner and peripherally induced Treg are depleted through the Fcγ receptor signaling pathway, so anti-CTLA-4 nanobodies lacking the Fc structural domain can promote anti-tumor responses without inducing colitis. This may lay the foundation for subsequent immunotherapy (65). In summary, PD-1/PD-L1 inhibitors and CTLA-4 inhibitors may enhance T cell activation and proliferation, thereby mediating the development of colitis.

#### Cytokines imbalance

Cytokines have been shown to be important mediators of intestinal inflammation (66, 67), of which Th1 and Th17 are highly involved in the pathogenesis of intestinal inflammation (68). Among them, Th17 cells are able to secrete IL-17 and IL-22, which potently inhibit the expression of chemokines, block the infiltration of pro-inflammatory immune cells into the colon, and make the body resistant to colitis and colon cancer (69). IL-6 is a cytokine critical for the differentiation of naive T cells into Th17 cells (70), in patients with ICI-mediated colitis, the expression level of IL-6 in the intestine is higher than that in normal intestinal tissue. IL-6 blockade can increase the density of CD4 + CD8 + effector T cells, thereby enhancing the anti-tumor effect (71). It can also reduce the number of Th17 cells, macrophages and myeloid cells, which have pro-inflammatory or inhibitory anti-tumor immunity. Its reduction alleviates the inflammatory response and relieves immunosuppression (72). IL-22, SCF Stem Cell Factor, an important cytokine, were all elevated in serum samples from patients with colitis. These all suggest a role for cytokines in ICI-mediated colitis (73). Patients with ICI-mediated colitis exhibited mucosal Treg expansion, increased CD8 tissue-resident memory T cells expressing CXCL13 and Th17, increased ITGB2-expressing CD8 T cells and GNLY-expressing cytotoxic CD4 T cells in the systemic circulation, and increased vascular endothelial cells expressing hypoxia-related genes (32). TNF-like cytokine 1A (TL1A) and its receptor DR3 are also upregulated in ICI-mediated colitis (74). DR3-mediated activation of Innate lymphoid cells-3 can lead to intestinal inflammation (75). In addition, baseline IL-17 levels are associated with the development of colitis, while the combination of baseline TGF-β1 and IL-10 levels is associated with clinical outcomes after ipilimumab treatment (76). IL-17A also enhances PD-L1 expression and promotes anti-PD-1 therapeutic resistance by modulating the P65/NRF1/miR-15b-5p pathway, and blocking IL-17A improves anti-PD-1 efficacy in microsatellite-stabilized MSS colorectal cancer mouse models (77). IL-23 expression was upregulated in the sera of patients who developed irAEA after combination immunotherapy with anti-CTLA-4 and anti-PD-1, and its elevation was positively correlated with the severity of irAEs. Simultaneous use of anti-IL-23 antibodies in combination immunotherapy relieves colitis while maintaining antitumor efficacy (78). High concentrations of CXCL9 and CXCL10 are associated with the risk of irAE (79). Another study on the combined immunotherapy of patients with melanoma with CTLA-4 and PD-1 showed that IL-1β and TNF were overexpressed in the gut compared to normal tissue (80). It has been reported that TNF-α cooperates with IFN-γ to regulate intestinal epithelial homeostasis (81). All of the above studies suggest that cytokines act as important mediators and cooperate with T cells to participate in the occurrence and progression of ICI-mediated colitis.

#### Intestinal microbial imbalance

Recent evidence suggests that the composition of a patient’s gut microbiota significantly affects the risk of ICI-mediated colitis, although this effect is not yet fully understood (82). Several studies have discussed the relationship between microbiota characteristics and ICI-mediated colitis, revealing differences in the composition of gut microorganisms between responders and non-responders. However, results often differ and conflict regarding which specific microorganisms are associated with colitis.

Administration of F. prausnitzii reshaped the gut microbiota composition, alleviated ICI-induced colitis, and simultaneously enhanced antitumor immune responses (83). Patients with colitis exhibit reduced diversity within the first 10 days of illness, increased Proteobacteria and Veillonella, and decreased Faecalibacterium. patients exhibit only acute, transient disorders early in the course of colitis and return to normal before recovery from colitis (84). A 16S rRNA sequencing analysis of fecal samples from FMT responders revealed significantly increased gut microbial α-diversity post-FMT, elevated abundances of Collinsella and Bifidobacterium, and reduced CD8+ T cell counts (24). Gut microbiome analysis (via 16S rRNA sequencing) in metastatic melanoma patients receiving ipilimumab showed that increased abundance of Bacteroidetes, Rikenellaceae, and Barnesiellaceae was associated with reduced risk of colitis (85). A similar study found that patients with ICI-induced colitis harbored abundant Faecalibacterium and other Firmicutes (86). Mice enriched with B. fragilis exhibited reduced incidence of irAEs following exposure to anti-CTLA-4 inhibitors (87). Existing studies demonstrate that distinct bacterial types correlate with ICI-induced colitis. Research on antibiotic use, fecal transplantation, and the microbiome in relation to ICI-mediated colitis remains in the preclinical phase, indicating significant unexplored potential in this field.

## Pharmacotherapy

**Figure 12:**
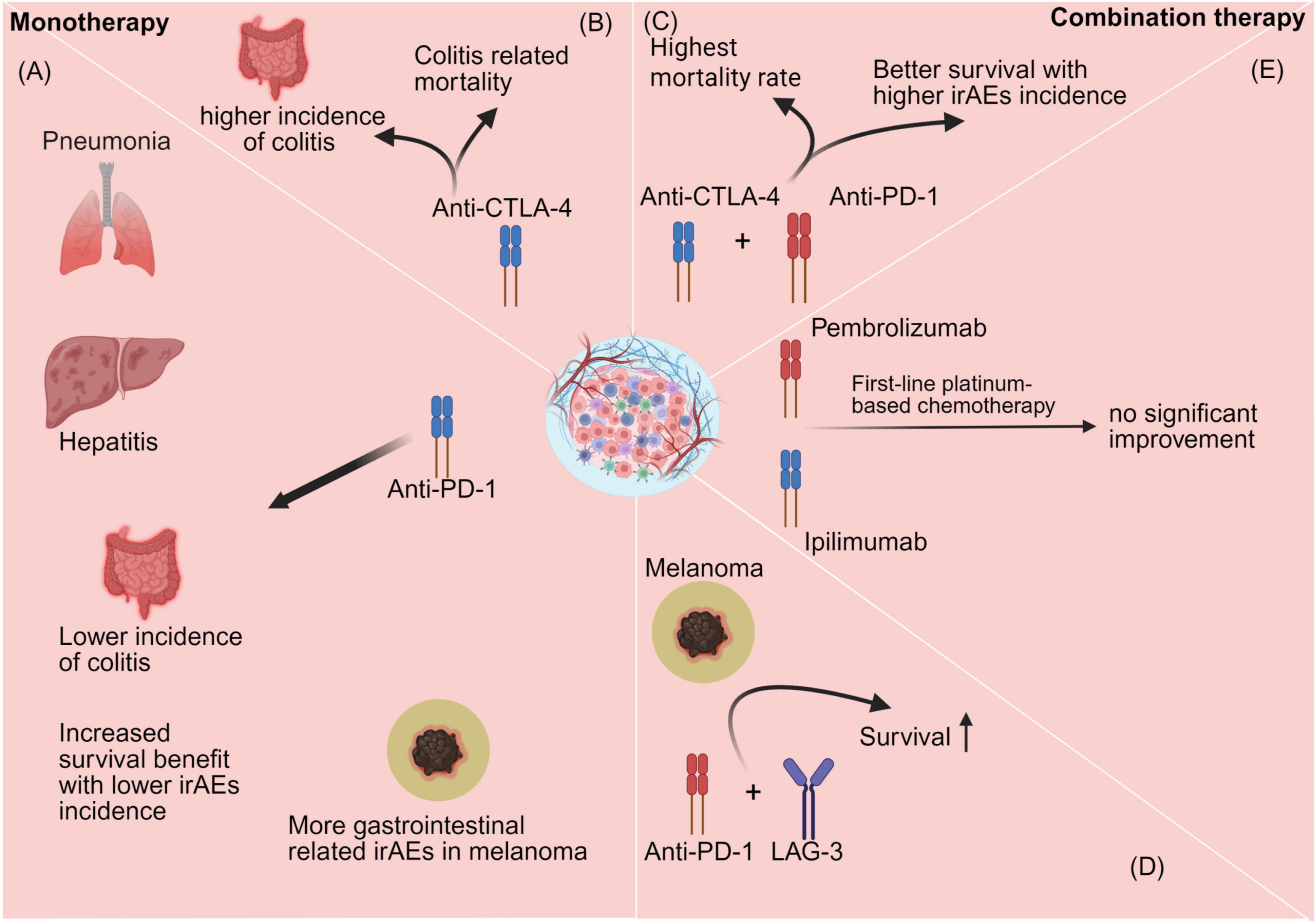
Different types of immune checkpoint inhibitors induce distinct patterns of immune-related adverse events. **(A)** Anti-PD-1 monotherapy is generally associated with a higher incidence of pneumonitis and hepatitis, but with fewer fatal cases of colitis compared with other treatment regimens, suggesting a relatively favorable safety profile. In melanoma, anti-PD-1 therapy has also been associated with an increased risk of gastrointestinal immune-related adverse events. **(B)** Anti-CTLA-4 therapy is more frequently associated with colitis and is linked to higher overall mortality. **(C)** Combined anti-PD-1 and anti-CTLA-4 blockade improves survival outcomes but is also accompanied by increased rates of immune-related adverse events and treatment-related mortality. **(D)** Combined anti-PD-1 and LAG-3 inhibition is similarly associated with improved survival outcomes. **(E)** By contrast, no significant therapeutic benefit has been observed when anti-PD-1 or anti-CTLA-4 therapy is combined with first-line platinum-based chemotherapy.

Current therapeutic strategies for ICI primarily involve monotherapy and combination immunotherapy. Different treatment approaches often lead to distinct immune-related adverse event profiles. Current combination regimens include ICIs co-administered with other immune checkpoint inhibitors, molecularly targeted agents, chemotherapy, or locoregional therapies (88). Here, we mainly focus specifically on monotherapy and ICI-based combination therapy with other immune checkpoint agents (**Figure 12**).

### Monotherapy

Compared with traditional chemotherapy, anti-PD-1 therapy such as pembrolizumab demonstrates longer progression-free survival and lower rates of grade 3 or higher adverse events and serious adverse events (89). In this study, pembrolizumab exhibited durable antitumor activity and fewer treatment-related adverse events, supporting its efficacy as a treatment for metastatic colorectal cancer with high microsatellite instability or mismatch repair deficiency.

However, different types of ICIs often result in irAEs of varying severity. For instance, monotherapy with anti-CTLA-4 agents carries a higher incidence of colitis and overall mortality, while monotherapy with anti-PD-1/PD-L1 is more strongly associated with pneumonia and hepatitis (90). Wang et al. found that toxicity-related mortality rates varied across treatment regimens. PD-1/PD-L1 plus CTLA-4 combination therapy exhibited the highest mortality rate at 1.23%, followed by anti-CTLA-4 (1.08%) and anti-PD-L1/PD-1 (0.36-0.38%). Among 193 anti-CTLA-4-related deaths, most typically resulted from colitis (n=135, 70%), whereas Anti-PD-1/PD-L1-related deaths are more strongly associated with pneumonia, hepatitis, and neurotoxicity, with colitis accounting for only a minority of cases (91). Robert et al. noted pembrolizumab demonstrated advantages over ipilimumab, including longer median overall survival and median progression-free survival, lower hazard ratios, lower incidence of grade 3–4 adverse events and grade 1–4 serious adverse events, and lower incidence of colitis (92). Khoja et al. also confirmed these findings (93). Notably, among the three most studied tumors in PD-1 trials—melanoma, non-small cell lung cancer, and renal cell carcinoma—melanoma exhibited a higher incidence of gastrointestinal irAEs.

### Combination therapy

Following numerous studies detailing the efficacy and adverse reactions of monotherapy with ICIs, combination immunotherapy involving anti-PD-1 and anti-CTLA-4 agents has gradually emerged as a leading approach. In 2015, combination immunotherapy received regulatory approval (94). In a Phase II clinical trial involving previously untreated patients with advanced melanoma, researchers observed that patients receiving the combination of Nivolumab and Ipilimumab demonstrated significantly higher 2-year overall survival rates compared to those treated with Ipilimumab monotherapy. However, this combination therapy is often associated with higher rates of Grade 3–4 adverse events and severe Grade 3–4 treatment-related adverse events. The most common adverse event in combination therapy is colitis, whereas monotherapy typically only causes diarrhea (95). In another phase III clinical trial for advanced melanoma patients, researchers found that patients receiving the combination of nivolumab and ipilimumab demonstrated longer median overall survival and median progression-free survival, along with lower hazard ratios for death and progression-free survival compared to monotherapy with either nivolumab or ipilimumab alone. Similarly, the combination therapy resulted in a higher proportion of grade 3–4 adverse events (96, 97). In 2022, researchers introduced Opdualag, a Relatlimab/nivolumab combination, as the first FDA-approved LAG-3 antibody (98). In advanced melanoma patients, nivolumab plus Opdualag demonstrated significantly improved PFS, higher OS, and greater ORR compared to nivolumab monotherapy (99). Furthermore, Powles et al. found that adding pembrolizumab to first-line platinum-based chemotherapy did not significantly enhance efficacy compared to chemotherapy alone (100). Another study on small cell lung cancer also failed to demonstrate an advantage of ipilimumab combined with platinum, even showing a higher treatment-related discontinuation rate (101). Nevertheless, the combination of ICIs with other therapies warrants further exploration. Currently, the high toxicity rates and significant efficacy of combination therapies have become a research hotspot in this field. When choosing between combination and monotherapy, researchers must balance the therapeutic effects of combination treatment against the incidence of irAEs. Furthermore, Wolchok et al. found that the efficacy and adverse reactions of ipilimumab in advanced melanoma patients exhibit a dose-dependent relationship (102). Perhaps researchers could further explore combination dosing to identify a balance point between treatment response and irAE incidence.

## Clinical utility and practice implications

The publication trends identified in this study can be linked to several recognizable shifts in clinical practice. Early highly cited studies were dominated by toxicity recognition in the ipilimumab era, whereas later bursts increasingly aligned with guideline-driven management of immune-mediated diarrhea and colitis. This transition is consistent with the publication of expert recommendations that formalized grading, corticosteroid initiation, endoscopic evaluation in selected cases, and escalation to infliximab or vedolizumab in refractory disease (23, 25). More recent translational studies on fecal microbiota transplantation and biomarker-guided monitoring suggest that the field is moving from general toxicity control toward individualized management of steroid-refractory colitis and microbiota-informed intervention (26). Thus, the scientometric trends observed here may be interpreted not only as changes in publication activity, but also as reflections of evolving clinical priorities and therapeutic decision points.

Beyond guideline-based management, the contemporary ICI-colitis literature increasingly supports the existence of a structured therapeutic pipeline for steroid-refractory disease. In this setting, selective immunosuppressive therapy has evolved from empirical escalation toward more defined therapeutic stratification, with infliximab and vedolizumab emerging as the two most prominent biologic options in current practice (23, 25). A two-center observational study further showed that both agents were effective in inducing clinical remission in immune-mediated diarrhea and colitis, but differed in time to clinical response, hospitalization burden, and recurrence patterns, suggesting that second-line treatment selection may carry distinct clinical trade-offs rather than representing interchangeable escalation options (103). Importantly, this therapeutic branch is now being prospectively formalized: the ongoing phase I/II randomized trial NCT04407247 directly compares infliximab and vedolizumab in patients with immune checkpoint inhibitor-related colitis, indicating that the field is moving from retrospective experience toward trial-based optimization of second-line management (104).

A second translational pipeline centers on microbiota restoration. FMT initially introduced as a rescue strategy for refractory disease, has now gained stronger translational support as a microbiota-directed therapeutic platform. Early work demonstrated that FMT could induce remission in refractory ICI-related colitis and was accompanied by reconstruction of the gut microbiota and increased Treg-associated features in the colonic mucosa (26). A subsequent study further validated microbiome alteration via FMT as an effective strategy in refractory immune-mediated colitis and linked treatment response to specific microbial signatures, thereby strengthening the rationale for microbiota-guided intervention (24). This concept has now progressed into formal clinical development, with multiple registered trials evaluating FMT in immune checkpoint inhibitor-mediated diarrhea/colitis, including NCT04038619, NCT04706611, and NCT06206707 (105–107). Overall, these studies suggest that microbiota-based therapy is gradually evolving from case-based use into a more systematic research direction.

A third translational pipeline links mechanistic discovery to biomarker development. Recent high-impact translational studies have shown that checkpoint colitis is characterized by epithelial barrier dysfunction, epithelial–immune crosstalk, and expanded pathogenic T-cell states, thereby defining candidate cellular and molecular axes for therapeutic targeting. Single-cell transcriptomic and spatial analyses have further clarified that both epithelial injury programs and distinct mucosal immune-cell populations contribute to disease pathogenesis, supporting a mechanism-to-target framework rather than a purely descriptive inflammatory model (32, 55). In parallel, clinically actionable biomarkers are beginning to emerge. Anti-integrin αvβ6 autoantibodies have been proposed as a biomarker for ulcerative colitis-like, severe ICI-colitis and may help identify patients requiring intensified immunosuppression (29). Taken together, these developments indicate that the ICI-colitis field is increasingly organized not only by toxicity recognition and management, but also by trial-ready therapeutic branches and mechanism-informed translational programs (**Table 8**).

**Table 8:** Representative clinical trial mapping and translational pipelines in ICI-colitis.

Field	Representative evidence	Translational implication	Key references
Steroid-refractory management	Infliximab and vedolizumab have become the major selective immunosuppressive options in current practice; a head-to-head randomized trial is ongoing	Moves second-line treatment from retrospective experience toward prospective comparative optimization	(23, 25, 103, 104)
Microbiota restoration	FMT rescue case series, translational validation in refractory disease, and multiple registered FMT trials	Establishes microbiota-directed therapy as a distinct interventional pathway rather than salvage-only anecdotal use	(26, 105–107, 141)
Mechanism to target pipeline	Single-cell and spatial studies identify epithelial barrier dysfunction, pathogenic T-cell states, and epithelial–immune crosstalk	Supports biologically stratified target discovery and mechanism-based intervention	(32, 57)
Colitis-specific biomarker pipeline	Anti-integrin αvβ6 autoantibodies and related mucosal inflammatory signatures	Links phenotype, severity stratification, and treatment-intensity decisions	(29)

## Biomarkers

While PD-L1, TMB, and MSI-H/dMMR remain important biomarkers for predicting response to immune checkpoint inhibitors, our keyword and thematic analyses suggest that biomarker research in ICI-colitis is increasingly moving toward noninvasive indicators of intestinal inflammation and markers linked to the gut microbiota and mucosal immune environment. Although ICIs induce relatively durable antitumor responses in various cancer patients, they also have drawbacks, including low response rates and the potential for severe adverse reactions. Therefore, selecting patients who can benefit from them is particularly important, and the identification of biomarkers plays a crucial role in enhancing the therapeutic efficacy of ICIs. Currently, biomarkers approved by the U.S. FDA for predicting ICI efficacy include PD-L1 expression, tumor mutational burden (TMB), mismatch repair deficiency (dMMR)/high microsatellite instability (MSI-H), and combination predictive biomarkers (108). Additionally, gut microbiota composition demonstrates considerable potential as a novel predictive biomarker.

### PD-L1

It is expected that PD-L1 should be expressed for anti-PD-L1 or anti-PD-1 therapy to be effective. Therefore, immunohistochemical detection methods targeting PD-L1 protein expression have been developed for clinical application. Currently, PD-L1 IHC is the first predictive biomarker for anti-PD-1 therapy in NSCLC patients approved by the FDA since 2015 (109). In this Phase I clinical trial of pembrolizumab for advanced NSCLC patients, researchers noted that PD-L1 expression in at least 50% of tumor cells correlated with improved efficacy (110). Furthermore, studies on metastatic urothelial carcinoma, metastatic clear cell renal cell carcinoma, and melanoma (111) confirmed PD-L1’s predictive value for patient response (112, 113). However, despite PD-L1’s widespread use, its overall diagnostic accuracy remains low. Andrew A. Davis et al. analyzed primary studies associated with 45 FDA drug approvals from 2011 to April 2019, finding PD-L1 predictive value in only 28.9% of cases, with non-predictive cases accounting for 53.3% (17).

### MSI-H/dMMR

In 2017, the FDA approved pembrolizumab for treating unresectable or metastatic solid tumors in MSI-H/dMMR patients, marking the first FDA-approved anti-tumor therapy based on a biomarker. However, the prevalence of MSI-H varies across different cancers, ranging from 0% to 16.5% (114). MSI refers to the failure of DNA mismatch repair mechanisms during DNA replication, leading to alterations in microsatellite sequence length (115). MSI has been identified in patients with multiple types of malignant tumors and serves as a critical indicator for prognosis and treatment response (116, 117). Based on MMR status, tumors are classified as dMMR or pMMR. Tumors with loss of MMR gene expression (including MSH2, MLH1, MSH6, or PMS2) are defined as dMMR, while others are classified as pMMR. Despite the promising potential of MSI/dMMR as a biomarker for ICI immunotherapy, a significant number of patients fail to benefit from it (118). Diagnostic errors introduced by testing methods represent a significant contributing factor (119). Current detection approaches include IHC, PCR, and next-generation sequencing (NGS). NGS serves as the third alternative method for MSI measurement, enabling the determination of MSI by quantifying altered microsatellite lengths. This approach does not require normal tissue as a control, imposes lower demands on sample quality, and offers greater compatibility compared to PCR. NGS can simultaneously provide information on MSI loci, MMR gene status, and other genetic states. Furthermore, NGS enables MSI monitoring using peripheral blood samples. However, high costs, complex data analysis processes, and the absence of unified evaluation standards limit the widespread clinical adoption of NGS (120). Currently, a more valuable approach should be proposed.

### TMB

TMB represents the total number of non-synonymous somatic mutations per megabase in the coding regions of a tumor genome. Tumors with high TMB often exhibit greater immunogenicity through the production of neoantigens (121). Alexandrov et al. demonstrated that TMB serves as a biomarker for predicting response to immunotherapy (122). Furthermore, a study examining TMB in advanced solid tumor patients receiving pembrolizumab monotherapy found that 29% of the tTMB-high group achieved objective responses, compared to only 6% in the non-tTMB-high group. The incidence of colitis was 2/790. While ensuring safety and efficacy, this suggests that high tissue TMB may serve as an effective predictive biomarker for response to pembrolizumab monotherapy in patients with recurrent or metastatic advanced solid tumors who have previously received treatment (123). Furthermore, significant associations between TMB and ICI response have been reported across tumor types including urothelial carcinoma (124), SCLC (125), NSCLC (126), and melanoma (127). This suggests TMB serves as a biomarker predicting patient response across multiple tumor types. Currently, two approaches exist for TMB assessment: WES typically considers non-synonymous somatic mutations, while NGS generally employs a more comprehensive method encompassing both synonymous and non-synonymous single nucleotide variants (108).

### Gut microbiota as a potential biomarker

The gut microbiota also holds immense potential as a biomarker. Extensive research has confirmed its role in predicting the efficacy of immunotherapy for tumors such as melanoma, gastrointestinal cancers, and lung cancer.

Gut microbiome diversity has been shown to correlate with immunotherapy efficacy. For instance, in melanoma patients receiving anti-PD-1 therapy, responders exhibited significantly increased α-diversity in their gut microbiome and higher relative abundance of Ruminococcaceae within the gut (128). Furthermore, higher microbial diversity was associated with improved immunotherapy response in NSCLC patients receiving anti-PD-1 therapy (129) and in renal cell carcinoma patients treated with anti-PD-1 (130). However, several studies involving larger cohorts failed to confirm this association (131, 132).

On the other hand, specific types of gut bacteria are also associated with responses to ICI therapy. Each research team identified distinct bacterial signatures linked to different immunotherapy responses, which may be related to multiple factors or because function rather than specific species determines treatment efficacy (133). For example, in advanced melanoma patients receiving ipilimumab, baseline gut microbiota rich in Faecalibacterium genus and other Firmicutes rather than Bacteroides correlated with favorable clinical responses and ipilimumab-induced colitis (86). Conversely, another study on ipilimumab-treated metastatic melanoma found increased abundance of Bacteroidetes phylum Bacteroides—particularly B. thetaiotaomicron and B. fragilis—correlated with resistance to ICI-induced colitis (85, 87). These studies demonstrate entirely opposite outcomes. Furthermore, elevated Akkermansia muciniphila abundance in responders to anti-PD-1 therapy for hepatocellular carcinoma and NSCLC suggests this bacterium may serve as a potential microbial biomarker for predicting ICI efficacy (134, 135). However, another study proposed that antibiotic use does not appear to influence nivolumab efficacy in NSCLC, showing no effect on progression-free survival (136). In summary, no single bacterium can serve as a consistently reliable biomarker across studies. Future research should incorporate larger sample sizes and consider the crosstalk between clinical characteristics and gut microbiota in immunotherapy (137).

Beyond utilizing gut microbiota as biomarkers, therapeutic approaches targeting gut microbiota are currently under investigation. For example, a study treating ICI-induced refractory colitis via fecal microbiota transplantation (FMT) found complete resolution of clinical symptoms post-FMT, reduced inflammation, ulcer healing, significantly decreased CD8+ T cell density, and a relative increase in CD4+ FoxP3+ T cell density. This suggests FMT-mediated gut microbiome regulation is associated with marked and rapid improvement in refractory ICI-related colitis (26).

### Akkermansia

The value of Akkermansia muciniphila (Akk) in ICI-related colitis is more appropriately understood as that of a potential microbiota-based biomarker with translational promise, rather than a single reliable marker that has already been fully validated. Current evidence indicates that Akk is closely linked to ICI efficacy and host microbial ecological status. In a prospective validation study including 338 patients with advanced NSCLC, baseline fecal Akk was associated with a higher objective response rate and longer overall survival, and this association remained independent of PD-L1 expression, antibiotic exposure, and performance status. Notably, however, an excessively high abundance of Akk (>4.799%) was instead associated with poorer prognosis, leading the authors to propose that Akk may more likely reflect a signal related to intestinal barrier injury/repair status, rather than functioning as a simple biomarker (27). Meanwhile, cross-cohort metagenomic analyses in melanoma showed that although Akk, together with Roseburia spp. and Bifidobacterium pseudocatenulatum, could form part of a responder-associated microbial panel, no single bacterial species could serve as a completely stable biomarker across different cohorts, suggesting that Akk must be interpreted within the broader microbial ecological context (137). Furthermore, an integrated study of 996 patients from seven clinical cohorts found that structural variants of Akkermansia muciniphila were associated not only with ICI efficacy but also with irAEs, indicating that its potential value may extend beyond efficacy prediction to include stratification of toxicity susceptibility (138). However, more recent evidence also suggests that this association is highly context-dependent: in NSCLC patients with low PD-L1 expression, tumor tissue positivity for Akk was paradoxically associated with worse progression-free survival, implying that the biological significance of Akk is not consistent across different sample types, abundance ranges, and host backgrounds (139). Therefore, in the context of ICI-related colitis, Akk is more appropriately regarded as a component of a composite biomarker framework, to be used for the integrated assessment of colitis susceptibility, intestinal mucosal barrier status, and the balance between antitumor benefit and immune-related toxicity.

### Fecal calprotectin

Within the more mature evidence framework for inflammatory bowel disease, fecal calprotectin (FC) has been established as a noninvasive inflammatory biomarker that can, in selected settings, partially substitute for endoscopic assessment. The 2023 AGA guideline states that, in patients with ulcerative colitis, FC can be used for disease monitoring and therapeutic decision-making; in particular, during symptomatic remission, an FC level <150 μg/g may help exclude active inflammation and reduce the need for routine endoscopic evaluation (140). This evidence base provides an important reference point for its translational application in ICI-related colitis. In the context of ICI-related colitis, the AGA Clinical Practice Update has recommended early monitoring of fecal inflammatory markers, including FC, in patients with grade 2 or higher diarrhea/colitis and even in selected milder cases, to assist with risk stratification, endoscopic decision-making, and disease monitoring (23). More direct evidence comes from the study by Zou et al., in which FC levels in 77 patients with immune-mediated diarrhea and colitis declined significantly after treatment and correlated with endoscopic severity and histologic activity. In that study, FC ≤116 μg/g predicted endoscopic remission, whereas FC ≤80 μg/g predicted histologic remission, suggesting that FC not only reflects intestinal inflammatory burden but may also serve as a dynamic marker of treatment response and deep remission (28). Furthermore, the ASCO guideline on immune-related adverse events has identified follow-up endoscopic evidence of mucosal healing, or FC ≤116 μg/g, as one of the potential parameters for guiding the timing of immune checkpoint rechallenge (25). Taken together, the available evidence supports FC as one of the most promising noninvasive biomarkers with clinical translational potential in ICI-related colitis, with utility for monitoring intestinal inflammatory activity, assessing treatment response, and, to some extent, reducing the need for repeated endoscopy. However, the current direct evidence is still derived mainly from retrospective studies, and future prospective, multicenter studies are still needed to validate the stability of its cutoff values and define its clinical applicability more clearly.

Beyond current colitis-focused biomarkers, future work should also consider modern blood-based and multi-omic approaches. Longitudinal ctDNA kinetics, peripheral T-cell repertoire reshaping, plasma proteomics, and extracellular vesicle profiling are increasingly being explored as minimally invasive tools to monitor immunotherapy response and immune-related toxicities in real time. Although these approaches have not yet been validated specifically for ICI-colitis, they represent an important translational direction for integrating tumor response, systemic immune dynamics, and toxicity surveillance within a single biomarker framework.

## Strengths and limitations

This study provides a dedicated bibliometric mapping of the ICI-colitis field and highlights how this literature has evolved from toxicity recognition toward clinically and translationally relevant themes, including steroid-refractory disease, mucosal immunology, microbiota-directed intervention, and biomarker development. However, several limitations should be considered. First, this study was based primarily on WoSCC, with PubMed used as a complementary validation source. Because bibliographic databases differ in journal coverage, indexing logic, and metadata structure, our map should be interpreted as a representative rather than exhaustive portrait of the ICI-colitis literature. In particular, clinically oriented gastroenterology and translational immunology studies may be captured differently across databases. Future scientometric studies in this field would benefit from multi-database integration. Additionally, the restriction to English-language publications may have led to the exclusion of non-English literature. Second, the use of different software tools for quantitative data analysis might have introduced minor discrepancies in the results. Another limitation is that the quality of the included studies was not systematically assessed. Future research could incorporate quality evaluations of selected literature to better understand the impact of studies on ICI-mediated colitis. Finally, data cleaning involved merging keywords with similar meanings, a step we believe enhances the quality and accuracy of this study. Despite these limitations, our findings provide valuable insights for researchers. Moreover, this study identifies emerging research foci and trends in the field, which may help improve the quality and impact of future publications and facilitate more efficient exploration of potential research directions. Finally, because the present study was based primarily on co-occurrence, co-citation, and burst analyses of bibliographic metadata, it should be interpreted as a descriptive map rather than a causal model of knowledge development. Future work may benefit from integrating citation-network analysis, bibliographic coupling, multilayer network inference, and causal machine learning approaches, particularly when linked to full-text content, clinical trial data, and multi-omic biomarker datasets.

## Conclusion

This study analyzed the trends and hotspots in research related to ICI-mediated colitis. We anticipate that future research will primarily focus on the following directions. First, elucidating the mechanisms underlying ICI-mediated colitis and other irAEs is essential for the development of novel therapeutic agents. Second, with the emergence of new ICIs, combination strategies involving chemotherapy, radiotherapy, or other ICIs warrant further investigation. Third, identifying patients who are most likely to benefit from ICI therapy remains a critical challenge, underscoring the need for continued exploration and validation of predictive biomarkers. Rather than functioning solely as a descriptive scientometric summary, this study outlines how the ICI-colitis literature has evolved across recognition, management, and translational phases. These findings may help frame future work around biologically stratified toxicity research, clinically actionable biomarkers, and microbiota-informed therapeutic strategies.

## Data Availability

The original contributions presented in the study are included in the article/**Supplementary Material**. Further inquiries can be directed to the corresponding authors.
