# Supplementary material for: Research landscape, thematic evolution, and translational insights of immune checkpoint inhibitor-induced colitis: a bibliometric analysis (2006-2025)
Source: Front Immunol. 2026 May 18;17:1817557. doi: 10.3389/fimmu.2026.1817557 (PMC13223026; doi:10.3389/fimmu.2026.1817557)

**Search formula**

("Immune Checkpoint Inhibitors"[Mesh] OR "immune checkpoint inhibitor*"[Title/Abstract] OR "checkpoint inhibitor*"[Title/Abstract] OR "anti-PD-1"[Title/Abstract] OR "anti-PD-L1"[Title/Abstract] OR "anti-CTLA-4"[Title/Abstract] OR ipilimumab[Title/Abstract] OR nivolumab[Title/Abstract] OR pembrolizumab[Title/Abstract] OR atezolizumab[Title/Abstract] OR durvalumab[Title/Abstract] OR avelumab[Title/Abstract] OR cemiplimab[Title/Abstract] OR relatlimab[Title/Abstract] OR (("PD-1"[Title/Abstract] OR "PD-L1"[Title/Abstract] OR "CTLA-4"[Title/Abstract]) AND (blockade[Title/Abstract] OR inhibitor*[Title/Abstract] OR antibod*[Title/Abstract]))) AND ( "Colitis"[Mesh] OR colitis[Title/Abstract] OR "immune-related colitis"[Title/Abstract] OR "immune-mediated colitis"[Title/Abstract] OR "checkpoint inhibitor-induced colitis"[Title/Abstract] OR "checkpoint inhibitor colitis"[Title/Abstract])

**Data screening process**

**
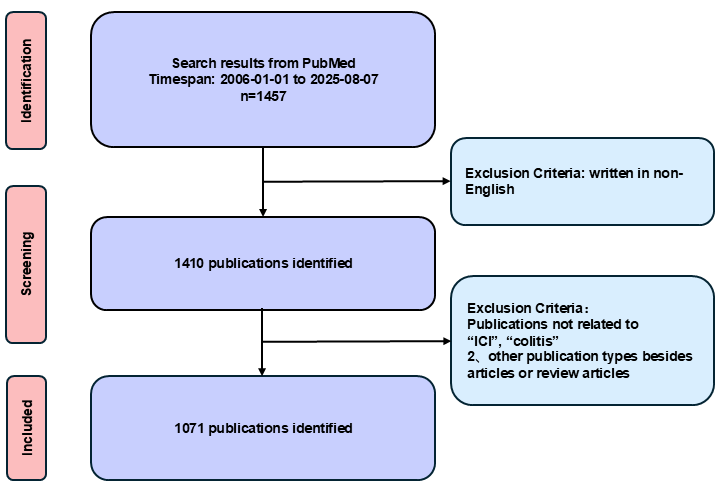
**

**Structural validation**

To further assess the robustness of the bibliometric findings beyond record counts alone, we performed a cross-database structural validation using PubMed under aligned restrictions for time span, language, and document type. Rather than using PubMed simply as a numerical supplement to WoSCC, we treated it as a sensitivity check to determine whether the major knowledge structures identified in the WoSCC-based analysis were reproducible across databases. Specifically, we compared the two datasets at four levels, including core journals, leading authors, high-frequency keywords, and trend topics.

**Journal validation**

At the journal level, we compared the main publication outlets identified in WoSCC and PubMed. We found that the two databases showed a broadly similar journal distribution, with only modest differences. Such discrepancies are acceptable, as PubMed has a stronger indexing orientation toward biomedical literature.


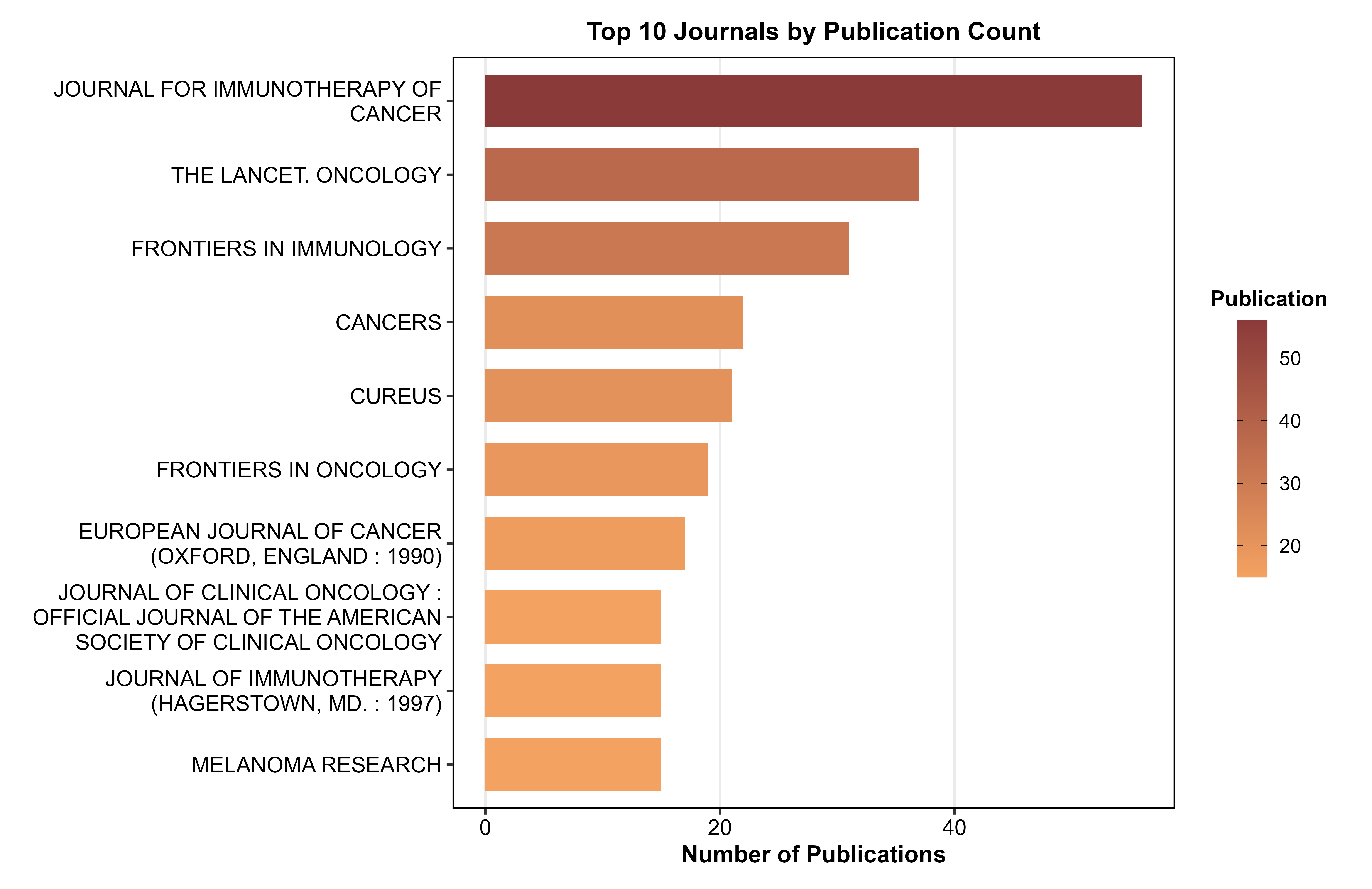


**Author validation**

At the author level, we found a high degree of consistency in both highly productive authors and highly cited authors, indicating that the principal academic contributors in this field were captured with reasonable robustness.


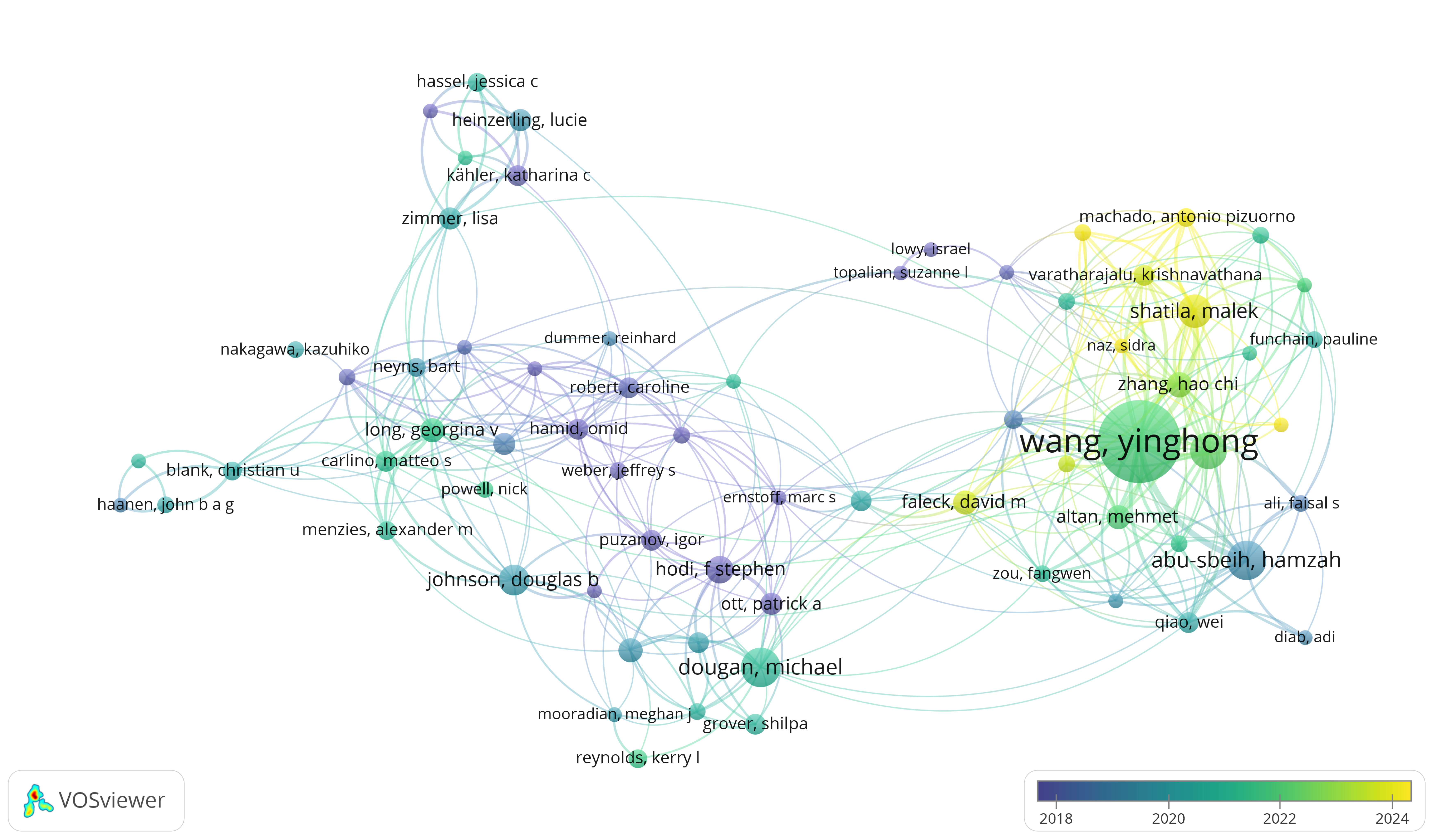


**Keyword validation**

At the keyword level, we likewise found that recent research has continued to focus on topics such as the gut microbiota, clinical trials, and CTLA-4 antigen.


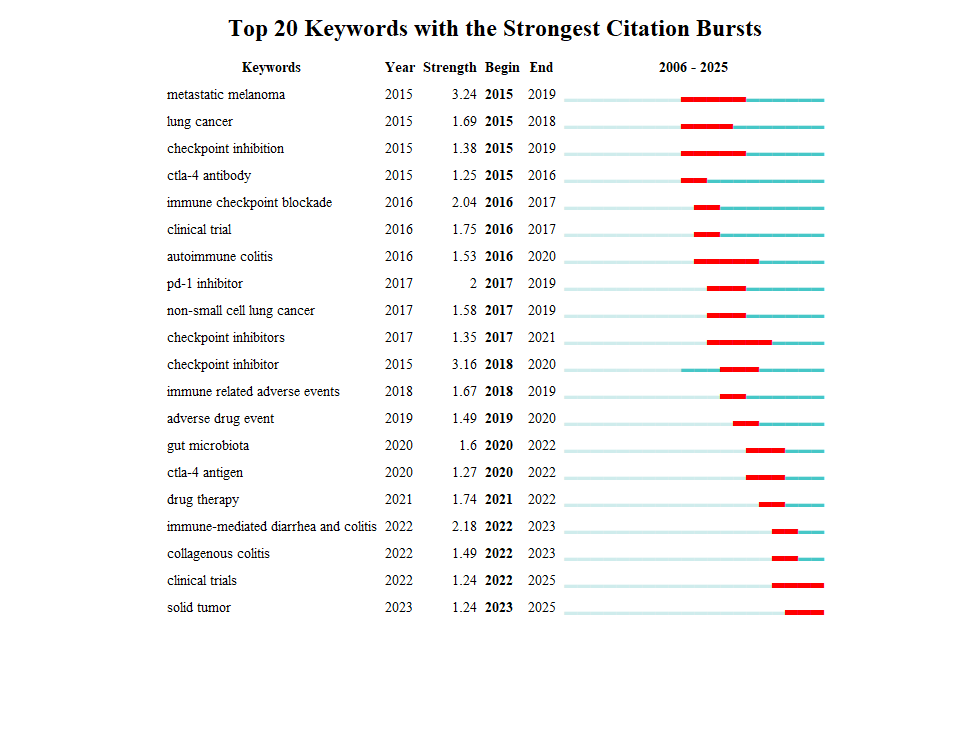


**Trend topic validation**

At the trend-topic level, we observed some differences between the results obtained from the two databases. Further analysis is still needed to clarify the underlying reasons for these discrepancies.


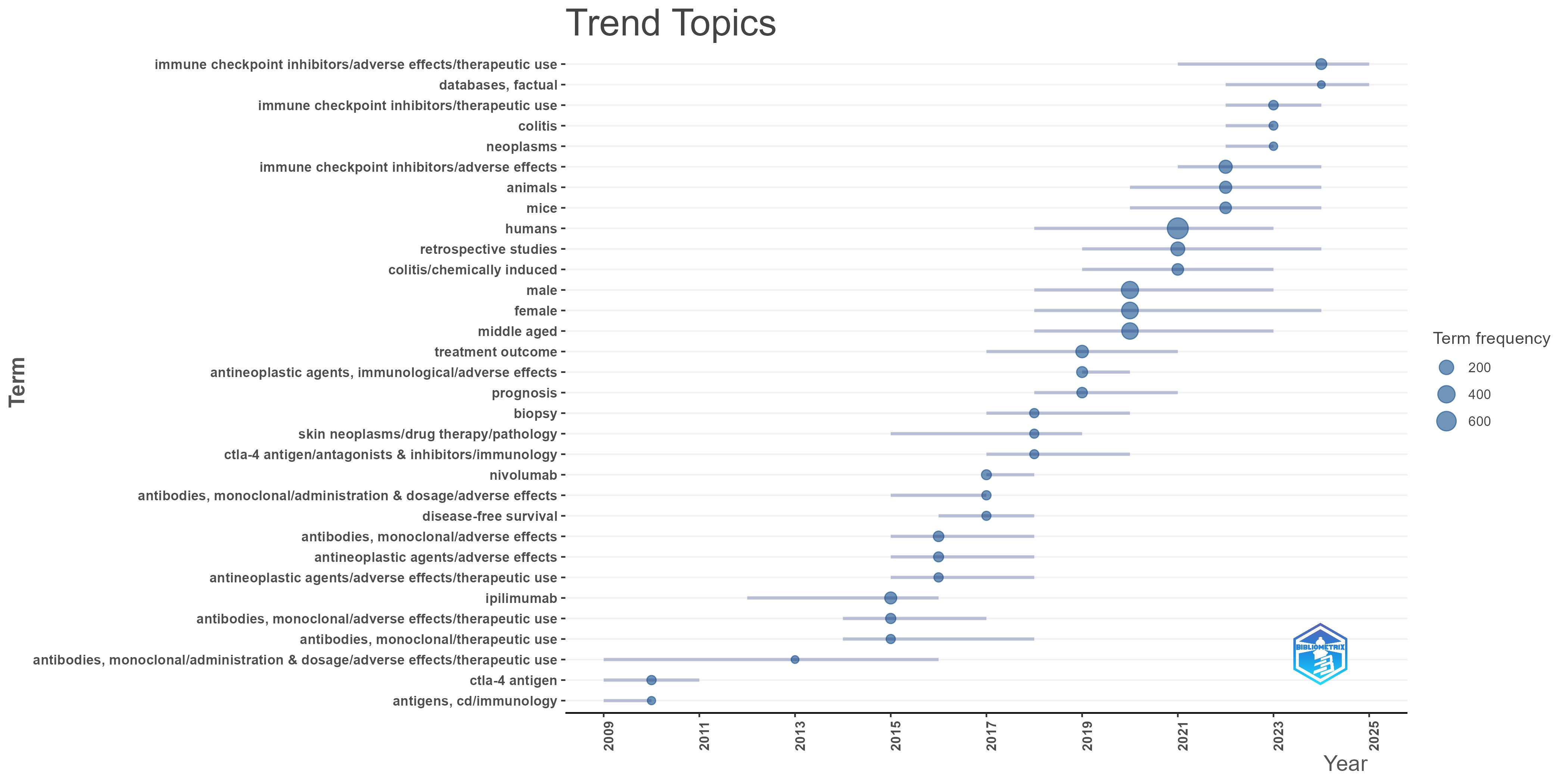

Supplement: Supplementary file 1 [file Table1.docx]
